# Guideline-conform translation and cultural adaptation of the Addenbrooke’s Cognitive Examination III into German

**DOI:** 10.3205/000280

**Published:** 2020-04-06

**Authors:** Björn Weiss, Julius J. Grunow, Max Rosenthal, David Hilfrich, Rudolf Mörgeli, Bruno Neuner, Friedrich Borchers, Antje Kraft, Henning Krampe, Claudia Denke, Claudia D. Spies

**Affiliations:** 1 Department for Anesthesiology and Operative Intensive Care Medicine (CCM, CVK), Charité – Universitätsmedizin Berlin, corporate member of Freie Universität Berlin, Humboldt Universität zu Berlin, and Berlin Institute of Health, Berlin, Germany; 2 Vision and Motor System Research Group, Department of Neurology, Charité – Universitätsmedizin Berlin, corporate member of Freie Universität Berlin, Humboldt Universität zu Berlin, and Berlin Institute of Health, Berlin, Germany

**Keywords:** neurocognitive disorders, neuropsychological test, translation and cultural adaptation, Addenbrooke's Cognitive Examination III

## Abstract

**Objective:** Age-related disorders, such as dementia, significantly contribute to the global burden of disease. Adequate screening in the primary care setting is critical for early detection and proper management. The Addenbrooke’s Cognitive Examination III (ACE-III) is an open-source neuropsychological test with superior diagnostic quality in comparison to the Mini-Mental State Examination (MMSE). Our aim was to perform a guideline-conform English-German translation and cultural adaptation of the ACE-III in order to enable implementation in German-speaking countries.

**Methods:** The translation and cultural adaptation were performed in accordance with the “Principles of Good Practice for the Translation and Cultural Adaptation Process for Patient-Reported Outcomes (PRO) Measures” from the International Society for Pharmacoeconomics and Outcome Research (ISPOR) (Wild et al. 2005). Four separate English-German translations were compiled into one German consensus translation, which was then translated back into English and compared to the original English version. After comparison, the German consensus translation was revised with emphasis on the identified differences between the English original version and the English translated version. This revised German consensus translation was subsequently evaluated for clinical applicability on a 5-point scale (0 – not applicable; 5 – applicable without any restrictions) by 20 practitioners experienced in the field of neuropsychological testing, using an anonymized, paper-based 22-item survey.

**Results:** Nineteen of the 20 practitioners (95.0%) rated the German ACE-III translation as overall applicable. The median rating was 4.0 [IQR (4.0/5.0)]. When evaluating survey items assessing the applicability of the individual 19 subtests of the ACE-III, all of them (100%) were rated as applicable with a median rating of 4.5 [IQR (4.1/4.9)].

**Conclusion:** The German ACE-III translation in its current form is generally applicable and can be utilized for clinical and scientific purposes.

## Zusammenfassung

**Zielsetzung:** Altersbedingte Erkrankungen wie Demenz nehmen eine immer zentralere Rolle in unserer alternden Gesellschaft ein und tragen maßgeblich zur globalen Krankheitslast bei. Ein adäquates Screening in der stationären und ambulanten Patientenversorgung ist eine der Hauptvoraussetzungen für die optimale Therapie von Demenzerkrankungen. Das Addenbrooke’s Cognitive Examination III (ACE-III) ist ein frei zugängliches neurokognitives Messinstrument mit sehr hoher diagnostischer Qualität, das dem Mini-Mental-Status-Test (MMST) überlegen ist. Ziel der Arbeit war es daher, eine leitliniengerechte englisch-deutsche Übersetzung und kulturelle Adaptation des ACE-III durchzuführen, um die Verwendung im deutschsprachigen Raum zu ermöglichen.

**Methoden:** Es wurde eine Übersetzung und kulturelle Adaptation im Einklang mit den „Principles of Good Practice for the Translation and Cultural Adaptation Process for Patient-Reported Outcomes (PRO) Measures” der International Society for Pharmacoeconomics and Outcome Research (ISPOR) (Wild et al. 2005) durchgeführt. Vier individuelle englisch-deutsche Übersetzungen wurden erstellt und in einer deutschen Konsensübersetzung vereinigt. Anhand dieser wurde anschließend eine Übersetzung zurück ins Englische erstellt und mit dem englischen Original verglichen. Nach dem Vergleich wurde die deutsche Konsensübersetzung mit speziellem Augenmerk hinsichtlich der Abweichungen zwischen dem englischen Original und der englischen Übersetzung revidiert. Die überarbeitete deutsche Konsensübersetzung wurde dann mittels eines anonymisierten, papierbasierten Fragebogens bestehend aus 22 Items von 20 Experten im Feld der neurokognitiven Testung auf einer Skala von 0 (nicht anwendbar) bis 5 (uneingeschränkt anwendbar) hinsichtlich der Anwendbarkeit beurteilt.

**Ergebnisse:** Neunzehn der 20 Befragten (95%) bewerteten die deutsche Version des ACE-III als insgesamt anwendbar. Der Median der Anwendbarkeitsbeurteilung war 4.0 [IQR (4.0/5.0)]. Im Hinblick auf Fragebogenitems, welche sich mit spezifischen Aspekten der Anwendbarkeit beschäftigen, wurden 19 der 19 Subtests (100%) als anwendbar eingeschätzt mit einer medianen Anwendbarkeitsbeurteilung von 4.5 [IQR (4.1/4.9)].

**Schlussfolgerung:** Die deutsche Version des ACE-III ist insgesamt anwendbar und kann für den Gebrauch im klinischen wie auch im wissenschaftlichen Rahmen empfohlen werden.

## Introduction

The significant improvement in life expectancy, especially in developed countries, has led to a rise in the occurrence of age-related disorders across all medical fields [1], [2]. Syndromes associated with cognitive dysfunction have increased both in incidence and prevalence, particularly in the older population [2]. The complex of clinically significant cognitive dysfunction encompasses the common forms of dementia, such as Alzheimer’s disease (AD) and frontotemporal dementia (FTD), but also less clinically obvious entities, such as mild cognitive impairment (MCI) [3], [4]. The clinical presentation of major neurocognitive disorders can show marked variation in deficits, resulting in distinguishable patterns of dysfunction, even at the stage of MCI. Patients in early stages of AD, for example, show a tendency for deficits in memory and spatial awareness, whereas FTD patients may present early with behavioral or speech-related impairments [3]. Early detection of cognitive impairment and a timely characterization of deficits can aid the clinician in the allocation of therapeutic resources tailored to the patient’s specific deficits, ultimately improving disease trajectory, and decreasing comorbidities. There is currently a lack of biomarkers for the early detection of dementia syndromes and cognitive dysfunction, and as such, neuropsychological testing remains the standard upon which clinical diagnostics are based [5]. Neuropsychological test batteries are central elements of the differential diagnostic process of cognitive impairment, and they allow for timely diagnostics of neurocognitive disorders and clinically significant cognitive dysfunction within the context of other disorders. Specialist neurocognitive testing serves as the gold standard for evaluation of cognition, requiring a clinician with specialized training and experience in the application of such tests [5]. This fact, coupled with the time-intensive nature of full-scale neuropsychological test batteries (typically 2–4 hours in length), has created a strong demand for cognitive screening tools. Such screening tools can be quickly administered, and since less training is usually required, these tests may be employed by a variety of non-specialized health care professionals. Screening tools identify patients with cognitive impairment with an acceptably high sensitivity and specificity, and possibly even aid in the differential diagnosis of types of neurocognitive disorders. The most commonly used of these screening tests is the Mini-Mental State Examination (MMSE) [6]. However, there is some evidence showing that the MMSE has low sensitivity in detecting mild cognitive impairment and dementias of the frontotemporal type, and some institutions argue that utilization is cost-prohibitive [7], [8], [9]. The first edition of the Addenbrooke’s Cognitive Examination (ACE) was developed to address some of the perceived deficits of the MMSE [3]. It contained all the basic elements of the MMSE – allowing a simultaneous calculation of the MMSE score – with a more extensive evaluation of language, memory and visuospatial abilities, while also adding verbal fluency testing. It has been shown that utilization of brief cognitive assessments has an inherent risk for misclassification, e.g. 21% for the MMSE and 16% for the Memory Impairment Screen [10].

The ACE and its successor, the ACE-Revised (ACE-R), have been validated in multiple languages and patient collectives, and while their overall sensitivity and specificity is comparable to the MMSE for the detection of dementias, they have increased sensitivity for mild cognitive impairment and frontotemporal dysfunctions [11]. The ACE-III is an improved version of the ACE-R, with an enhanced diagnostic accuracy in comparison to other brief cognitive examination tools, including the highest diagnostic accuracy in comparison with the MMSE, the Memory Impairment Screen, the Montreal Cognitive Assessment, and the Rowland Universal Dementia Assessment Scale for detection of Alzheimer’s Disease [12].

The ACE-III was developed to address certain weaknesses of its predecessors in the domains of comprehension, repetition and the visuospatial assessment [13]. Additionally, it has replaced certain elements that were originally found in the MMSE, clearing up issues of copyright facilitating a broader implementation of the test across cultural, geographical, and economic domains [14].

A year following the publication of the ACE-III, the Mini-ACE (M-ACE) was introduced [15]. It contains a limited number of items from the ACE-III questionnaire, and is intended as a shorter version for situations where application of the complete ACE-III test-battery is not feasible (e.g. in a busy primary care practice). The M-ACE was also shown to perform on-par with the MMSE in terms of diagnostic capabilities [16]. As it contains exclusively items that are also part of the ACE-III, its score can be calculated from it as well.

The aim of the project was to translate the ACE-III forms, including the user manual, into the German language, and adapt their contents to better fit the cultural particularities of the German-speaking countries. The translated version should enable local clinicians to utilize this tool in the cognitive work-up of their patients and enable further clinical validation.

In order to achieve reliable results, cognitive tests should be conducted in a familiar language and draw from the participant’s own cultural background. The ACE-III and all its predecessors were developed in English and for an English-speaking cultural background, which is not directly applicable to patients in Germany. To achieve applicability in a German context, a verbatim translation of the test contents does not suffice; rather, an appropriate adaptation of the test content to reflect the German cultural background is required.

## Methods

The German version of the ACE-III/M-ACE was developed according to the International Society for Pharmacoeconomics and Outcome Research (ISPOR) guideline [17], which had been established in order to harmonize and improve the translation and cultural adaptation process for patient-reported outcomes (PRO) measures. This guideline represents the gold standard for the translation and cultural adaptation of clinical examination tools, including neurocognitive tests, and encompasses 10 steps:

1. preparation,
2. English-German translation,
3. compilation into one German consensus translation,
4. translation back into English,
5. review of English translation,
6. harmonization,
7. cognitive debriefing,
8. review of cognitive debriefing results and finalization,
9. proofreading,
10. final report [17].

In the initial preparation step, the original authors (Hsieh et al. [12]) were approached regarding consent to perform a translation of the ACE-III/M-ACE into the German language. After approval, four separate English-German translations were performed by a physician and three further members of the research team. After finalization of the individual English-German translations, they were combined into one German consensus translation, following the discussion of incongruencies within the primary English-German translations and difficulties regarding the cultural adaptation. In order to achieve the best consensus, other published ACE-III translations were screened for potential approaches to solve these problems, and/or the original author was contacted [18], [19]. The translation from German back into English was performed by a physician who had not yet been involved in the project, who is a native speaker of both English and German, and who is also experienced in the field of neurocognitive testing. The translated English version was evaluated in comparison to the original ACE-III/M-ACE by the translation team in order to identify and correct any major differences, ambiguities or inaccuracies in the consented German translation. Afterwards, the author of the English original was asked to assess the translation back into English with a special emphasis on the elements that deviated from the original ACE-III/M-ACE. On the basis of the feedback from the original author, the German consensus translation of the ACE-III was revised and harmonized by the translation team.

This revised and harmonized version was subsequently evaluated for clinical applicability, using a cognitive debriefing process in the form of a survey (Attachment 1) between 1 and 31 March 2018. This anonymized, paper-based survey received approval by the ethics committee of the Charité – Universitätsmedizin Berlin (EA4/010/18). Participants consented to participate by returning the completed questionnaire. Within the Department of Anesthesiology and Operative Intensive Care Medicine (CCM, CVK), Charité – Universitätsmedizin Berlin, we included resident physicians, specialist physicians, senior physicians and medical students engaged in research projects ≥18 years of age who had experience in the field of neurocognitive testing.

The survey consisted of 22 items, including two items concerning the respondent’s position within the department and years of experience with neurocognitive tests. The other 20 items asked the respondent to rate the applicability of the ACE-III on a scale of 0 (not applicable) to 5 (applicable without restrictions). 19 survey items assessed the 19 subtests of the ACE-III, which refer to five cognitive domains: attention (3 subtests), memory (5 subtests), language (7 subtests), fluency (1 subtest) and visuospatial abilities (3 subtests). The final item asked the respondent to rate the overall applicability of the translated German version of the ACE-III as a whole.

A priori, it was defined that a score ≥4 would indicate that the subtest and/or the entire test was applicable. In case of an applicability score <4, the practitioner was asked to select the reason for insufficient applicability from a predefined list (content, language and practicability). The primary outcome was the percentage of respondents who rated the complete German translation of the ACE-III as applicable. Secondary outcomes were the percentage of subtests rated as applicable, median applicability of the entire translation, as well as frequency of limiting factors for the entire translation and specific subtests. The results of the cognitive debriefing were analyzed and discussed by the entire ACE-III team to identify potential areas of improvement or potential barriers to be addressed. Finalization was performed under consideration of these results, prior to proofreading and preparation of the final report. As the M-ACE is a reduced form of the ACE-III, its applicability was calculated with ratings from the ACE-III survey. Data are shown as median with interquartile ranges in case of continuous variables or as absolute count and percentage in case of categorical variables.

## Results

All ten steps of the “Principles of Good Practice for the Translation and Cultural Adaptation Process for Patient-Reported Outcomes (PRO) Measures” from the ISPOR [17] were carried out.

- **1. Preparation: **The author of the English original approved the translation of the ACE-III, M-ACE, as well as the ACE-III Manual into a German version.
- **2./3. English-German translation and compilation into one German consensus translation: **The individual primary English-German translation did not show any major discrepancies in terms of content. A few congruent uncertainties regarding the literal translation of a non-German neurocognitive test and the impact on its validity emerged during the consensus development (see ACE-III Supplement in Attachment 2 for detailed information).
- **4./5. Translation back into English and review of English translation:** The content of the English translation was congruent with the original English version. Differences were only identified in sections that had to be culturally adapted, which were not translated literally in order to sustain validity.
- **6. Harmonization:** After submitting the ACE-III and M-ACE to the author of the English original, the German translation was accepted and published on the website for use.
- **7./8. Cognitive debriefing, review of cognitive debriefing results, and finalization:** Initially, the survey was distributed among 30 qualified staff in the department. Of the recipients, 20 completed the survey within the predefined time frame. Three of the respondents did not give a response to one of the survey items (items 7, 15 and 21) yielding 437 of 440 completed data points and a response rate of 99.3% within the received surveys. The composition of the respondent cohort is depicted in Table 1 (Tab. 1). The overall median duration of experience with neurocognitive tests was 4.0 years (2.0–6.0).

**Figure 1:**
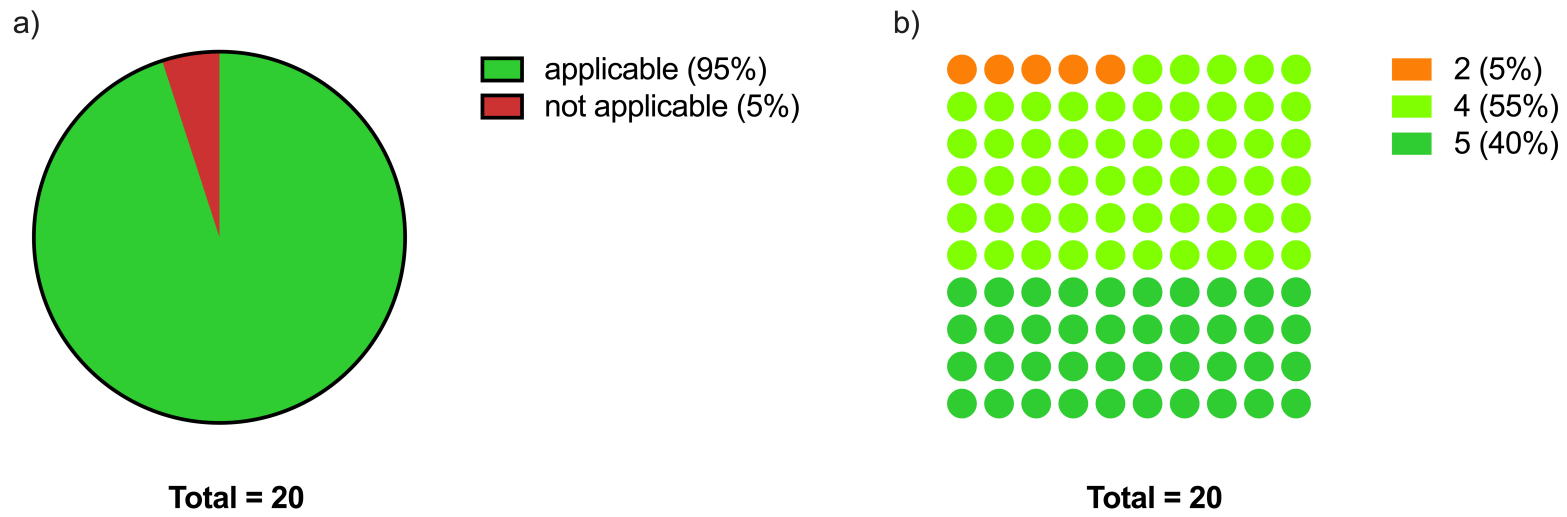
ACE-III applicability a) Dichotomised applicability of the German version of the ACE-III as a whole with a score ≥4 defined as applicable and <4 as not applicable b) Distribution of the applicability scores for the German version of the ACE-III as a whole

The German translation of the ACE-III as a whole was rated as applicable by 19 of 20 (95%) respondents, with a median applicability rating of 4.0 [IQR (4.0/5.0)] (Figure 1 (Fig. 1), Table 2 (Tab. 2)).

**Figure 2:**
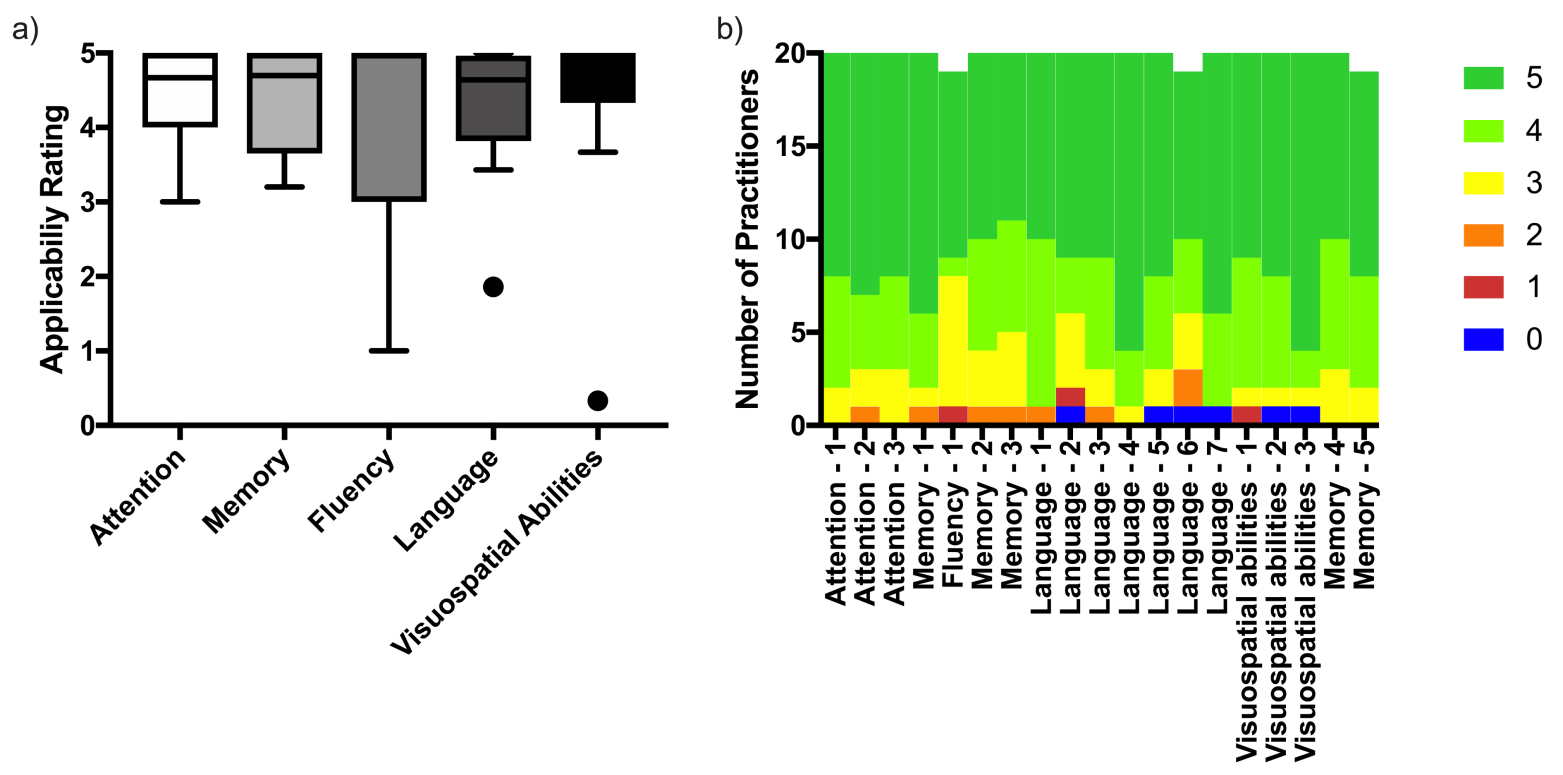
Item applicability a) Applicability rating of the different cognitive domains as median and interquartile range b) Distribution of the applicability scores for the subtests

The high applicability rating of the ACE-III as a whole was also reflected when separately assessing the five cognitive domains, as well as the 19 subtests, as they all yielded a median applicability rating ≥4 (Figure 2 (Fig. 2), Table 2 (Tab. 2)). The combined median applicability rating for all items was 4.5 [IQR (4.1/4.9)]. The applicability rating for the overall translation was dichotomized according to the a priori defined applicability cut-off value. The mean applicability rating for all items per respondent was dichotomized in the same manner. When comparing both these values (applicable n [%]: 19 [95] vs 15 [75]), no significant difference was present p=0.125).

**Figure 3:**
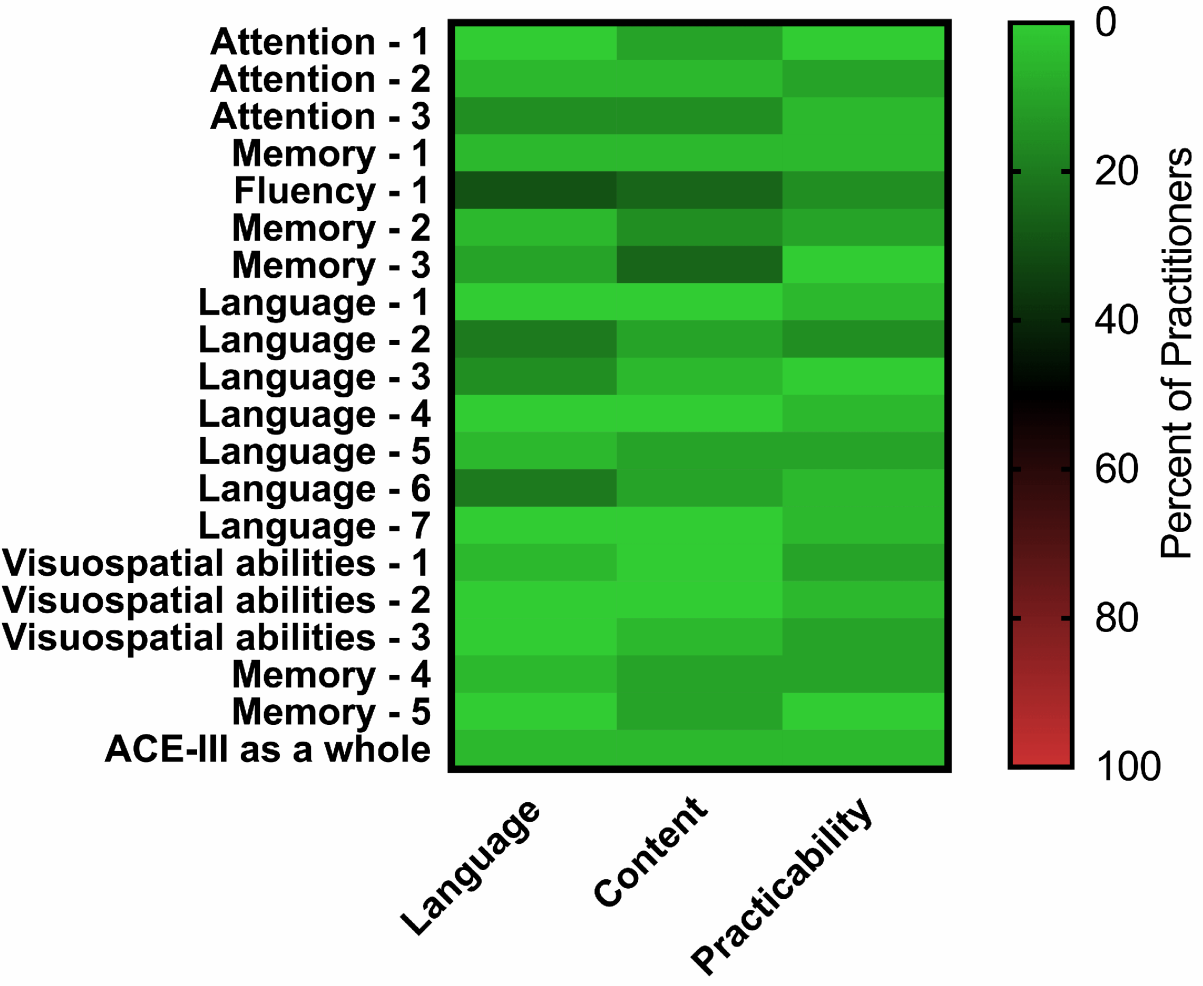
Distribution of limiting factors for the German version of the ACE-III as a whole as well as all subtests separately

One respondent (5%), who is currently a resident and has 4 years of experience with neurocognitive assessments, listed language, content and practicability as limiting factors for the applicability of the German version of the ACE-III as a whole (Figure 3 (Fig. 3)). Further information regarding the selected limiting factors for each item is shown in Figure 3 (Fig. 3).

The M-ACE presents similar applicability ratings as the ACE-III as a whole, with a median rating of 4.3 [IQR (3.8/5.0)].

## Discussion

This is the first systematic, guideline-conform German translation of the Addenbrooke’s Cognitive Examination III, allowing its implementation in German-speaking countries for cognitive examination and diagnostics, both for clinical routine and research purposes. During our cognitive debriefing with practitioners experienced in the field of neurocognitive testing, 95% rated the translation as overall applicable, confirming our primary hypothesis and giving us confidence in recommending its implementation in German-speaking countries. Furthermore, the interquartile range for the applicability rating for the ACE-III as a whole, as well as for almost all individual items, was above the a priori defined applicability cut-off score of 4, indicating a high agreement between practitioners.

Dementia has been recognized as a significant contributor to the global disease burden measured as disability-adjusted life years (DALYs). A major surge occurred between 1990 and 2010, almost solely attributable to the ageing of society [20]. Besides its impact on the individual, dementia also inflicts a significant economic burden on society, accounting for approximately $100 billion of formal health care costs, and an additional $100 billion of informal health care costs (defined as caregiving through spouses, family or friends during daily life), with an upward trajectory [21]. Therefore, the German clinical practice guideline for dementia recommends early diagnosis in order to enable timely management [22]. General practitioners have a central role in the screening and early detection of cognitive dysfunction, as they often represent the most frequent point of physician-patient contact, and are likely the initial contact person sought by patients (and relatives) confronted with cognitive impairment. Accordingly, the German clinical practice guideline for geriatric assessment recommends general practitioners to conduct a brief cognitive assessment in any patient presenting signs associated with dementia [23].

The Addenbrooke’s Cognitive Examination III is a neurocognitive assessment instrument used primarily for dementia screening. It has been shown to closely correlate with its predecessor (ACE-R), which had high diagnostic accuracy comparable to or better than the MMSE [24], [25]. The MMSE is the current standard for severity stratification and follow-up assessments of dementia patients [6].

The availability of a short and long version of the ACE-III is an additional benefit, allowing its application in a wider spectrum of settings and types of clinical encounter, e.g. using the short version for dementia screening in the primary care setting, and the long version for comprehensive cognitive assessment in the research setting. A further advantage of the ACE-III is its open-source availability, enabling its widespread utilization in settings with restricted budget, where the MMSE would be cost-prohibitive, e.g. general practitioners unwilling/unable to pay for individual screenings, or clinicians in low- and middle-income countries who could otherwise not afford routine testing [7]. Due to these advantages, we believe that the Addenbrooke’s Cognitive Examination III will be of great value for the assessment and management of dementia and other forms of cognitive dysfunction in German-speaking countries.

The translation of diagnostic tools and clinical stratification scores is prone to errors due to linguistic and cultural differences between the original and the target language, which in turn compromise their validity and usefulness in clinical practice. The original Addenbrooke’s Cognitive Examination III includes items testing an individual’s ability to understand and interpret figures of speech. In such cases, literal translation is possible, but not advisable, as a verbatim translation might not translate into a similarly common figure of speech in the target language. In such cases, more figurative forms of translation must be cautiously employed, as the translator’s interpretation directly influences the translation. The ISPOR has developed a composite guideline to implement a high-quality standard for translation and cultural adaptation of outcome measures [17]. We completed every step of the guideline recommendations during translation and validation to minimize the risk for bias. Nevertheless, there are multiple unvalidated and unsystematically translated versions of most diagnostic tools and clinical stratification scores, which severely impairs their usability for clinical and scientific purposes [26]. The nature of an increasingly globalized research community requires agreement on common patient measures, both across national borders and cultural spheres. A major issue with the international usability of gold-standard language-based tests is the fact that validation often takes place in a single language, usually in English. While this is beneficial to the research community – facilitating a common base of scientific communication – this reliance on English-language tests poses difficulties for clinicians in non-English speaking countries wishing to implement tests with an acceptable scientific basis. As our experience with the translation process shows, a sufficiently implementable translation of a complex neuropsychological test is a lengthy process, requiring input from numerous practitioners with a variety of skills, such as language proficiency, clinical expertise, experience with testing batteries of a similar format, among others.

In order to maintain scientific rigor and maximize widespread applicability of gold-standard tests, a high level of effort should be a mandatory step prior to implementation of such tests into practice.

A real-world validation of an adequately translated test should be the eventual goal of all translation endeavours. As such, future studies should address the validation of the German version of the Addenbrooke’s Cognitive Examination III for different fields of application, as well as the development of normative data. This would further improve the scientific value of the ACE-III in the German-speaking world.

Our study has some limitations. Firstly, we did not validate the translated ACE-III in an actual patient collective, nor did we develop normative data, as our aim was merely to develop a clinically applicable translation. As a result, validation of the translation on patients with specific disorders is the next step and should therefore be addressed in future studies. Secondly, our sample size is relatively small, although in the light of an interquartile range for overall applicability above our cut-off score of 4 (cut-off value for applicability) for the ACE-III as a whole, as well as for almost all individual items, we do not believe that our results are prone to be overly affected by the sample size.

## Conclusions

The translated and culturally adapted German version of the ACE-III as well as the M-ACE were shown to be applicable and can therefore be utilized during clinical routine and for research purposes.

## Notes

### Competing interests

The authors declare that they have no competing interests.

### Acknowledgement

We would like to thank Sharpley Hsieh for enabling and supporting us during the translation and cultural adaptation of the Addenbrooke’s Cognitive Examination III instrument.

### German ACE-III/M-ACE

- ACE-III A (German)
- ACE-III B (German)
- ACE-III C (German)
- M-ACE A (German)
- M-ACE B (German)
- M-ACE C (German)
- ACE-III Scoring (German)

## Supplementary Material

ACE-III Applicability Survey (German)

ACE-III Supplement

ACE-III A (German)

ACE-III B (German)

ACE-III C (German)

M-ACE A (German)

M-ACE B (German)

M-ACE C (German)

ACE-III Scoring (German)

## Figures and Tables

**Table 1:**
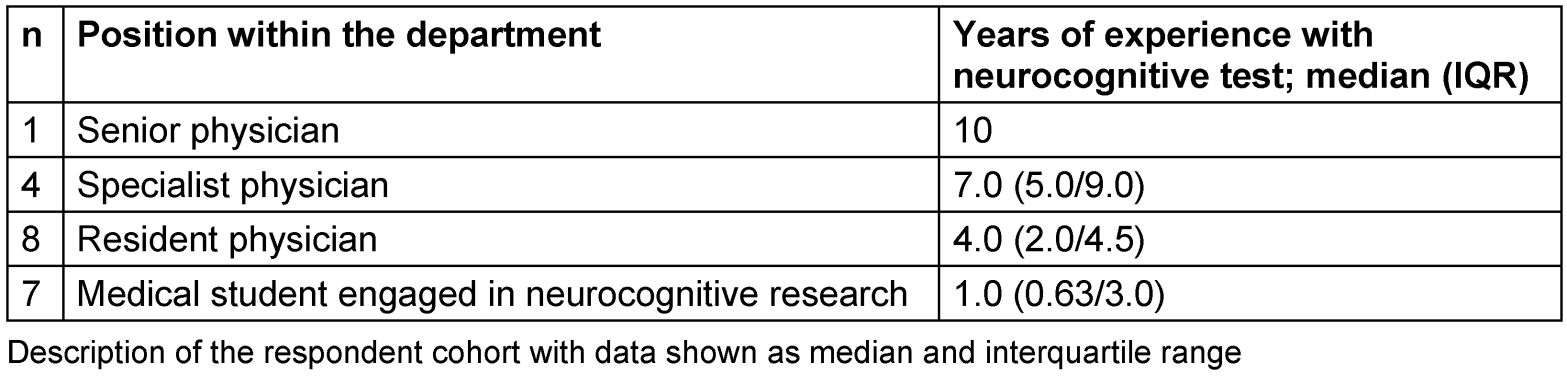
Respondent cohort

**Table 2:**
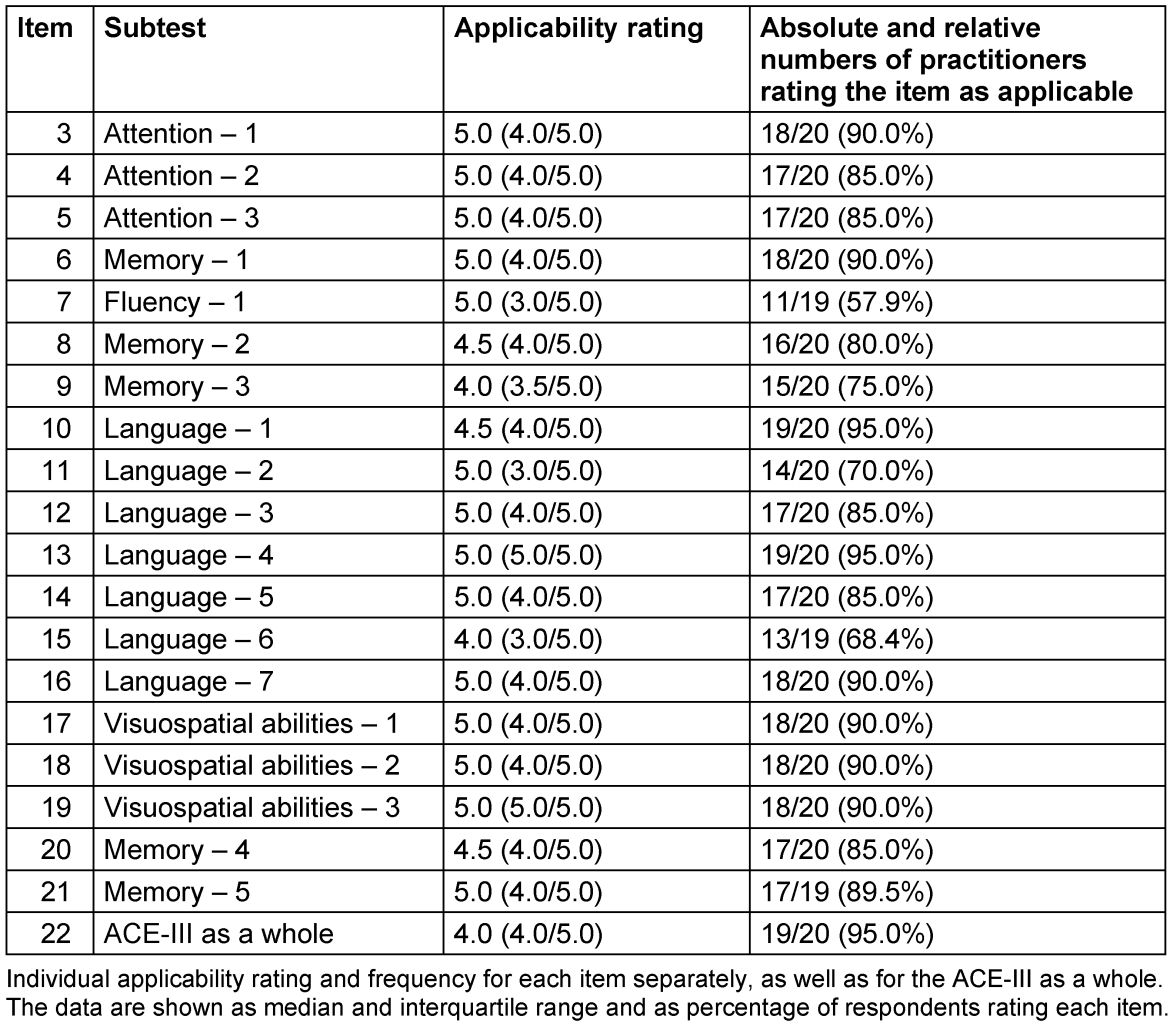
Individual applicability rating and frequency for all subtests, as well as for the ACE-III test as a whole; median (interquartile range) and number (%)
