# Supplementary material for: Guideline-conform translation and cultural adaptation of the Addenbrooke’s Cognitive Examination III into German
Source: Ger Med Sci. 2020 Apr 6;18:Doc04. doi: 10.3205/000280 (PMC7174851; doi:10.3205/000280)
Supplement: ACE-III Applicability Survey (German) [file GMS-18-04-s-001.pdf]

Kognitives Debriefing der leitliniengerechten Übersetzung des Addenbrooke's Cognitive Examination – III (ACE-III) Messinstruments

Sehr geehrte Mitarbeiterinnen, sehr geehrte Mitarbeiter,

im Rahmen der leitliniengerechten Übersetzung des Addenbrooke's Cognitive Examination – III (ACE-III) Messinstruments benötigen wir Ihre Hilfe. Der Übersetzungsprozess beinhaltet ein kognitives Debriefing zur Validierung der Anwendbarkeit des Messinstruments sowie zum Ausschluss einer Beeinflussung durch die Übersetzung und kulturelle Adaptation. Die Publikation des übersetzten Messinstruments, mitsamt kognitivem Debriefing, in einem peer-reviewed Journal wird angestrebt.

Im Folgenden würden wir Sie bitten jedes Item des ACE-III Messinstruments hinsichtlich der Anwendbarkeit in Bezug auf Sprache, Inhalt und Praktikabilität zu bewerten.

Die Items werden auf einer Skala von 0 (nicht anwendbar) bis 5 (uneingeschränkt anwendbar) beurteilt. Für jedes Item mit einem Punktewert < 4 würden wir Sie bitten die Faktoren zu nennen, welche die Anwendbarkeit einschränken.

Dieser Fragebogen kann in dem beiliegenden Umschlag verschlossen und ohne Absender bis zum 31.03.2018 in die im Sekretariat aufgestellten, abschließbaren Urnen eingeworfen werden. Diese werden mit Abschluss der Befragung durch die Projektgruppe „ACE-III“ geleert. Die Daten werden danach für die Auswertung elektronisch gespeichert und die Fragebögen nach der Dateneingabe vernichtet. Nach der Auswertung wird auch die elektronische Datenbank gelöscht.

Die Teilnahme an der Befragung ist freiwillig; über eine zahlreiche Beteiligung würden wir uns freuen. Ihre Anonymität bei der Befragung ist gewährleistet. Sollten Sie sich für oder gegen die Teilnahme an der Befragung entscheiden hat dies keinen Einfluss auf Ihre Beschäftigung. Die Befragung wurde dem Datenschutzbeauftragen der Charité – Universitätsmedizin Berlin sowie dem Gesamtpersonalrat gemeldet und von diesen, nach Durchsicht des papierbasierten Fragebogens sowie des geplanten Konzepts, genehmigt. Die Auswertung der Daten wird unter datenschutzrechtlichen Aspekten durch die Projektgruppe „ACE-III“ durchgeführt. Es wird keine personenbezogene Rückverfolgung bei der Teilnahme an der Befragung erfolgen. Die ausgefüllten Fragebögen werden nach dem Übertragen in eine elektronische Datenbank vernichtet. Die Daten werden nicht weitergegeben und nur für die oben beschriebenen Zwecke verwendet.

Verantwortliche für die Datenerhebung ist die Klinikdirektorin Univ.-Prof. Dr. med. C. Spies.

Vielen Dank für Ihre Mitarbeit!

**1 Welche berufliche Stellung haben Sie in unserer Klinik?**	
**Ärztlicher Dienst**	
**1.1**	☐ **Ärztliche/r MitarbeiterIn in Weiterbildung**
**1.2**	☐ **Fachärztin/Facharzt**
**1.3**	☐ **Oberärztin/Oberarzt**
**Andere Mitarbeiter**	
**1.4**	☐ **Studentischer MitarbeiterIn**

**2 Seit wie vielen Jahren arbeiten Sie mit neurokognitiven Messinstrumenten?**	
**2.1**	**Zeit in Jahren:**

3 Anwendbarkeit des Items

**AUFMERKSAMKEIT**						
➢ Fragen Sie: „Welche/n/s ... haben wir heute?"	Wochentag<br>______	Datum<br>______	Monat<br>______	Jahr<br>______	Jahreszeit<br>______	**Aufmerksamkeit** [Punkte 0-5] ☐
➢ Fragen Sie: „In welcher/m ... befinden wir uns?"	Zimmer/ Etage<br>______	Straße/ Krankenhaus<br>______	Stadt<br>______	Bundesland<br>______	Land<br>______	**Aufmerksamkeit** [Punkte 0-5] ☐

**3.1**	**Anwendbarkeit**
	☐ **0 (nicht anwendbar)** ☐ **1** ☐ **2** ☐ **3** ☐ **4** ☐ **5 (uneingeschränkt anwendbar)**
**3.2**	**Limitierender Faktor der Anwendbarkeit**
	☐ **Sprache** ☐ **Inhalt** ☐ **Praktikabilität**

4 Anwendbarkeit des Items

**AUFMERKSAMKEIT**	
➢ Sagen Sie: „Ich werde Ihnen drei Wörter sagen und bitte Sie, diese danach zu wiederholen: „Zitrone, Schlüssel, Ball". Nach erfolgter Wiederholung sagen Sie: „Versuchen Sie, sich diese Wörter zu merken, denn ich werde Sie später noch einmal danach fragen."<br>➢ Nur der erste Versuch wird bewertet.<br>➢ Wiederholen Sie bis zu drei Mal, wenn notwendig und notieren Sie die Anzahl der Versuche: _____	**Aufmerksamkeit** [Punkte 0-3] ☐

**4.1**	**Anwendbarkeit**					
	**☐ 0 (nicht anwendbar)**	**☐ 1**	**☐ 2**	**☐ 3**	**☐ 4**	**☐ 5 (uneingeschränkt anwendbar)**
**4.2**	**Limitierender Faktor der Anwendbarkeit**					
	**☐ Sprache**	**☐ Inhalt**	**☐ Praktikabilität**			

5 Anwendbarkeit des Items

**AUFMERKSAMKEIT**	
➢ Bitten Sie: „Ziehen Sie von der Zahl 100 jeweils 7 ab, bis ich Sie bitte aufzuhören."<br>➢ Unterbrechen Sie den Probanden bei einem Fehler nicht. Lassen Sie ihn fortfahren und überprüfen die folgenden Antworten (z.B. 93, 84, 77, 70, 63 – 4 Punkte).<br>➢ Stoppen Sie nach 5 Subtraktionen (93, 86, 79, 72, 65): ____ ____ ____ ____ ____	**Aufmerksamkeit** [Punkte 0-5] ☐

**5.1**	**Anwendbarkeit**					
	**☐ 0 (nicht anwendbar)**	**☐ 1**	**☐ 2**	**☐ 3**	**☐ 4**	**☐ 5 (uneingeschränkt anwendbar)**
**5.2**	**Limitierender Faktor der Anwendbarkeit**					
	**☐ Sprache**	**☐ Inhalt**	**☐ Praktikabilität**			

6 Anwendbarkeit des Items

**GEDÄCHTNIS**	
➢ Fragen Sie: „Welche drei Wörter hatte ich Sie gebeten zu wiederholen und sich dann zu merken?"<br>______ ______ ______	**Gedächtnis** [Punkte 0-3] ☐

**6.1**	**Anwendbarkeit**					
	**☐ 0 (nicht anwendbar)**	**☐ 1**	**☐ 2**	**☐ 3**	**☐ 4**	**☐ 5 (uneingeschränkt anwendbar)**
**6.2**	**Limitierender Faktor der Anwendbarkeit**					
	**☐ Sprache**	**☐ Inhalt**	**☐ Praktikabilität**			

7 Anwendbarkeit des Items

WORTFLÜSSIGKEIT

- **Buchstaben**

Sagen Sie: „Ich nenne Ihnen einen Buchstaben und möchte, dass Sie mir so viele Wörter wie möglich sagen, die mit diesem Buchstaben beginnen. Es dürfen aber weder Personen-, noch Ortsnamen sein. Nenne ich Ihnen beispielsweise den Buchstaben „K", wären „Katze, kaufen, kurz" , usw. mögliche Antworten, nicht jedoch „Katharina" oder „Kiel". Haben Sie das verstanden? Sind Sie bereit? Sie haben eine Minute Zeit. Bitte verwenden Sie den Buchstaben „P"."

Wortflüssigkeit [Punkte 0 – 7] ☐

≥ 18	7
14-17	6
11-13	5
8-10	4
6-7	3
4-5	2
2-3	1
0-1	0
Gesamt	korrekt

- **Tiere**

Sagen Sie: „Jetzt nennen Sie bitte so viele Tiere wie möglich. Alle Anfangsbuchstaben sind gestattet."

Wortflüssigkeit [Punkte 0 – 7] ☐

≥ 22	7
17-21	6
14-16	5
11-13	4
9-10	3
7-8	2
5-6	1
<5	0
Gesamt	Korrekt

7.1	**Anwendbarkeit**
	☐ **0 (nicht anwendbar)** ☐ **1** ☐ **2** ☐ **3** ☐ **4** ☐ **5 (uneingeschränkt anwendbar)**
7.2	**Limitierender Faktor der Anwendbarkeit**
	☐ **Sprache** ☐ **Inhalt** ☐ **Praktikabilität**

8 Anwendbarkeit des Items

GEDÄCHTNIS

- Sagen Sie: „Ich werde Ihnen einen Namen und eine Adresse sagen und möchte, dass Sie den Namen und die Adresse wiederholen. Damit Sie sich den Namen und die Adresse besser merken können, wiederholen wir sie drei Mal. Ich frage später noch einmal nach dem Namen und der Adresse."
- Nur der dritte Versuch wird gewertet.

Gedächtnis [Punkte 0 – 7] ☐

	Erster Versuch	*Zweiter Versuch*	*Dritter Versuch*
Peter Müller<br>Dorf Strasse 73<br>Wolfsburg<br>Niedersachsen			

8.1	**Anwendbarkeit**
	☐ **0 (nicht anwendbar)** ☐ **1** ☐ **2** ☐ **3** ☐ **4** ☐ **5 (uneingeschränkt anwendbar)**
8.2	**Limitierender Faktor der Anwendbarkeit**
	☐ **Sprache** ☐ **Inhalt** ☐ **Praktikabilität**

9 Anwendbarkeit des Items

GEDÄCHTNIS

- Name des/der amtierenden Bundeskanzlers/in...
- Name des/der amtierenden Bundespräsidenten/in..
- Name des/der amtierenden Präsidenten/in der USA..
- Name des US-amerikanischen Präsidenten, der in den 1960ern ermordet wurde..................................

Gedächtnis [Punkte 0 – 4] ☐

**9.1**	**Anwendbarkeit**					
	☐ **0 (nicht anwendbar)**	☐ **1**	☐ **2**	☐ **3**	☐ **4**	☐ **5 (uneingeschränkt anwendbar)**
**9.2**	**Limitierender Faktor der Anwendbarkeit**					
	☐ **Sprache**	☐ **Inhalt**	☐ **Praktikabilität**			

10 Anwendbarkeit des Items

SPRACHE

- Legen Sie einen Stift und ein Blatt Papier vor den Probanden. Zur Probe, bitten Sie ihn: „Heben Sie den Stift auf und dann das Blatt Papier." Falls dies nicht gelingt, geben Sie 0 Punkte und beenden Sie diesen Abschnitt.
- Wurde der Probeversuch korrekt durchgeführt, machen Sie mit den folgenden drei Aufgaben weiter:
  - "Legen Sie das Blatt Papier auf den Stift"
  - "Heben Sie den Stift auf, aber nicht das Blatt Papier"
  - "Reichen Sie mir den Stift, nachdem Sie das Blatt Papier berührt haben"
- Wichtig: Legen Sie Stift und Papier vor jeder Aufgabe erneut vor den Probanden.

Sprache [Punkte 0-3] ☐

**10.1**	**Anwendbarkeit**					
	☐ **0 (nicht anwendbar)**	☐ **1**	☐ **2**	☐ **3**	☐ **4**	☐ **5 (uneingeschränkt anwendbar)**
**10.2**	**Limitierender Faktor der Anwendbarkeit**					
	☐ **Sprache**	☐ **Inhalt**	☐ **Praktikabilität**			

11 Anwendbarkeit des Items

SPRACHE

- Sagen Sie: „Bitte schreiben Sie zwei Sätze. Der Inhalt der Sätze ist Ihnen überlassen. Ich möchte Sie allerdings bitten, in ganzen Sätzen zu schreiben und Abkürzungen zu vermeiden." Wenn dem Probanden kein Thema einfällt, können Sie Themen vorschlagen: „Sie könnten beispielsweise über Ihren letzten Urlaub, Ihre Hobbies, Ihre Familie oder Kindheit schreiben." Sollte der Proband nur einen Satz aufschreiben, bitten Sie um einen zweiten Satz.
- Jeder Satz muss sowohl Subjekt als auch Verb enthalten. Grammatik und Rechtschreibung werden bewertet. Die Sätze müssen nicht vom gleichen Thema handeln. Siehe Bewertungsanleitung für weitere Informationen.

Sprache [Punkte 0-2] ☐

**11.1**	**Anwendbarkeit**					
	☐ **0 (nicht anwendbar)**	☐ **1**	☐ **2**	☐ **3**	☐ **4**	☐ **5 (uneingeschränkt anwendbar)**
**11.2**	**Limitierender Faktor der Anwendbarkeit**					
	☐ **Sprache**	☐ **Inhalt**	☐ **Praktikabilität**			

12 Anwendbarkeit des Items

**SPRACHE**	
➢ Bitten Sie den Probanden folgende Wörter zu wiederholen: „Butterblume", „Ekzentriker", „unentzifferbar", „Statistiker"<br>➢ Vergeben Sie 2 Punkte wenn alle Wörter korrekt wiederholt wurden; 1 Punkt, wenn 3 der Wörter korrekt wiederholt wurden; 0 Punkte bei 2 oder weniger korrekten Wiederholungen.	**Sprache**<br>[Punkte 0-2]<br>☐

**12.1**	**Anwendbarkeit**					
	☐ **0 (nicht anwendbar)**	☐ **1**	☐ **2**	☐ **3**	☐ **4**	☐ **5 (uneingeschränkt anwendbar)**
**12.2**	**Limitierender Faktor der Anwendbarkeit**					
	☐ **Sprache**	☐ **Inhalt**	☐ **Praktikabilität**			

13 Anwendbarkeit des Items

**SPRACHE**	
➢ Bitten Sie den Probanden folgendes zu wiederholen: **„Es ist nicht alles Gold, was glänzt."**	**Sprache**<br>[Punkte 0-1]<br>☐
➢ Bitten Sie den Probanden folgendes zu wiederholen: **„Der frühe Vogel fängt den Wurm."**	**Sprache**<br>[Punkte 0-1]<br>☐

**13.1**	**Anwendbarkeit**					
	☐ **0 (nicht anwendbar)**	☐ **1**	☐ **2**	☐ **3**	☐ **4**	☐ **5 (uneingeschränkt anwendbar)**
**13.2**	**Limitierender Faktor der Anwendbarkeit**					
	☐ **Sprache**	☐ **Inhalt**	☐ **Praktikabilität**			

14 Anwendbarkeit des Items

SPRACHE

Sprache [Punkte 0-12]

➢ Bitten Sie den Probanden, folgende Bilder zu benennen:

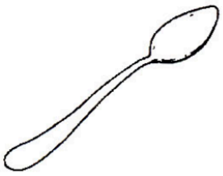
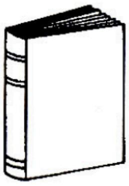
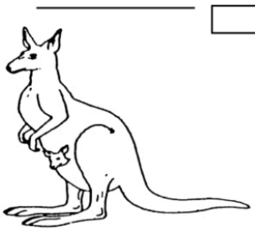
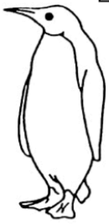
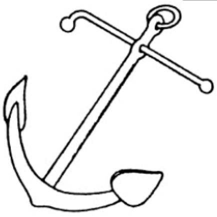
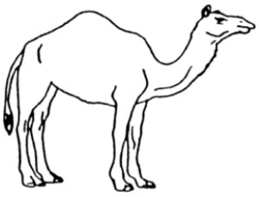
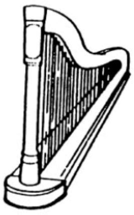
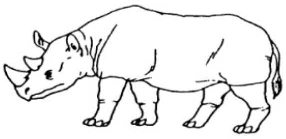
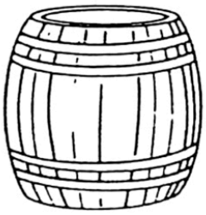
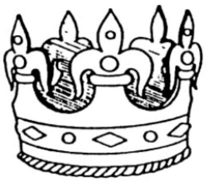
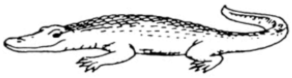
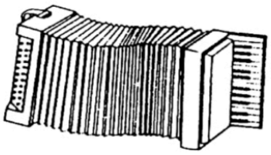

**14.1**	**Anwendbarkeit**					
	**☐ 0 (nicht anwendbar)**	**☐ 1**	**☐ 2**	**☐ 3**	**☐ 4**	**☐ 5 (uneingeschränkt anwendbar)**
**14.2**	**Limitierender Faktor der Anwendbarkeit**					
	**☐ Sprache**	**☐ Inhalt**	**☐ Praktikabilität**			

15 Anwendbarkeit des Items

SPRACHE	
➢ Mit Hilfe der oben gezeigten Bilder, bitten Sie den Probanden:<br>• Auf das Bild zu zeigen, das mit Monarchie in Verbindung gebracht werden kann ……………<br>• Auf das Bild zu zeigen, das ein Beuteltier darstellt ……………<br>• Auf das Bild zu zeigen, welches in der Antarktis gefunden werden kann ……………<br>• Auf das Bild zu zeigen, das einen Bezug zur Seefahrt hat ……………	**Sprache**<br>[Punkte 0-4]<br>☐

**15.1**	**Anwendbarkeit**					
	☐ **0 (nicht anwendbar)**	☐ **1**	☐ **2**	☐ **3**	☐ **4**	☐ **5 (uneingeschränkt anwendbar)**
**15.2**	**Limitierender Faktor der Anwendbarkeit**					
	☐ **Sprache**	☐ **Inhalt**	☐ **Praktikabilität**			

16 Anwendbarkeit des Items

SPRACHE	
➢ Bitten Sie den Probanden die folgenden Wörter zu lesen: (1 Punkt gibt es sofern alle Wörter korrekt sind)<br>**Uhr**<br>**Maß**<br>**fort**<br>**platt**<br>**Schrank**	**Sprache**<br>[Punkte 0-1]<br>☐

**16.1**	**Anwendbarkeit**					
	☐ **0 (nicht anwendbar)**	☐ **1**	☐ **2**	☐ **3**	☐ **4**	☐ **5 (uneingeschränkt anwendbar)**
**16.2**	**Limitierender Faktor der Anwendbarkeit**					
	☐ **Sprache**	☐ **Inhalt**	☐ **Praktikabilität**			

17 Anwendbarkeit des Items

VISUELL-RÄUMLICHE FÄHIGKEITEN

- Unendlichkeits-Symbol: Bitten Sie den Probanden, folgendes Symbol nachzuzeichnen.

Visuell-räumlich [Punkte 0-1] ☐

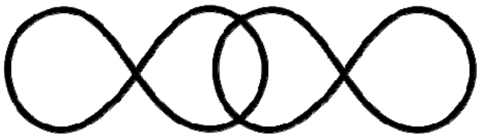

- Strichwürfel: Bitten Sie den Probanden, folgende Darstellung nachzuzeichnen (für die Punktevergabe, siehe Bewertungsanleitung).

Visuell-räumlich [Punkte 0-2] ☐

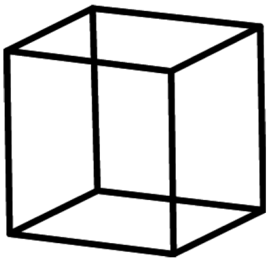

- Uhr: Bitten Sie den Probanden, das Ziffernblatt einer Uhr zu zeichnen, deren Zeiger auf Zehn nach Fünf stehen (für Punktevergabe, siehe Nutzerhandbuch: Kreis = 1, Nummern = 2, Zeiger = 2, sofern sie alle korrekt sind).

Visuell-räumlich [Punkte 0-5] ☐

**17.1**	**Anwendbarkeit**
	☐ **0 (nicht anwendbar)** ☐ **1** ☐ **2** ☐ **3** ☐ **4** ☐ **5 (uneingeschränkt anwendbar)**
**17.2**	**Limitierender Faktor der Anwendbarkeit**
	☐ **Sprache** ☐ **Inhalt** ☐ **Praktikabilität**

18 Anwendbarkeit des Items

**VISUELL-RÄUMLICHE FÄHIGKEITEN**	
➢ Bitten Sie den Probanden, die Punkte zu zählen, ohne auf sie zu zeigen.	**Visuell-räumlich**<br>[Punkte 0-4]

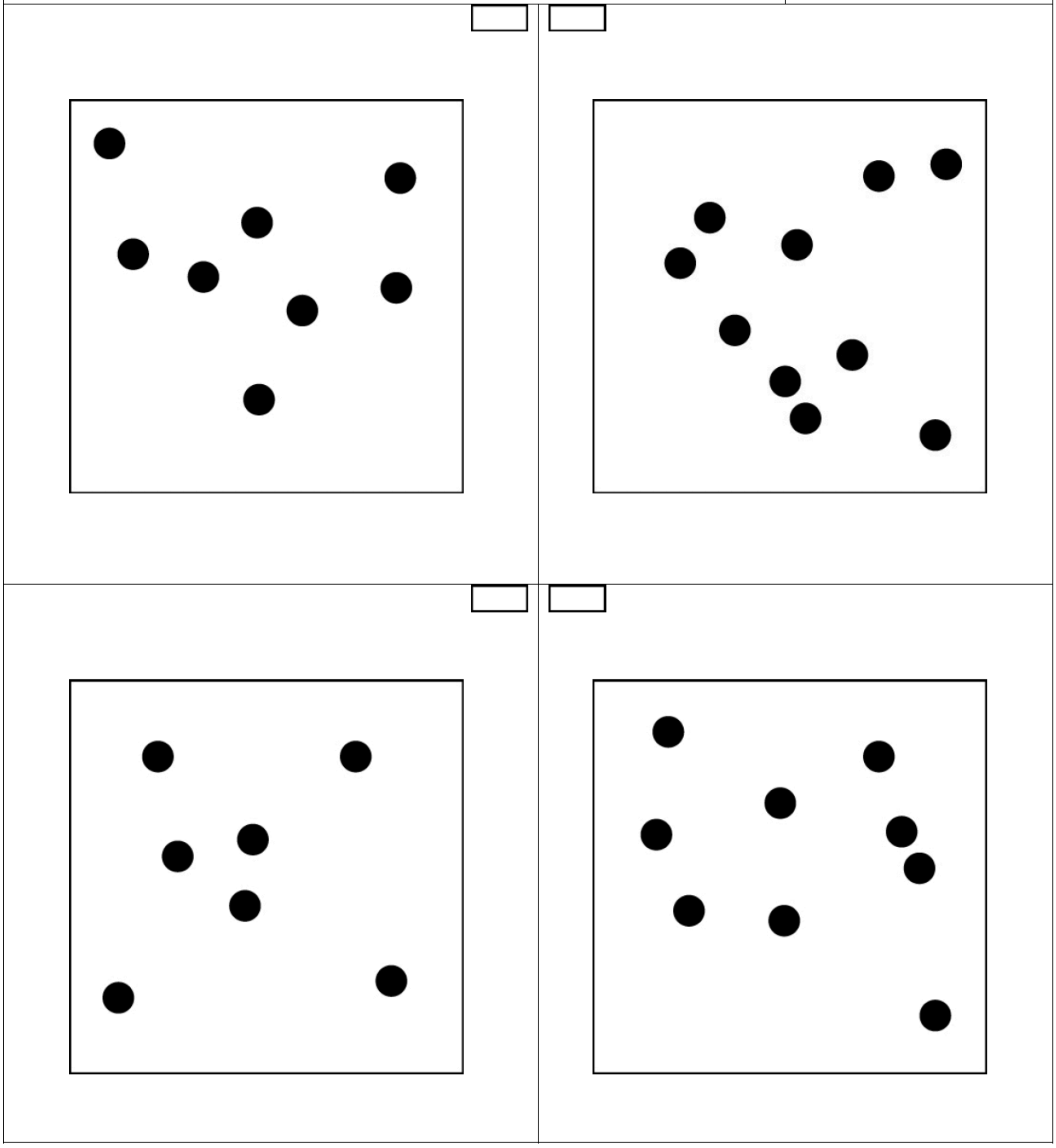

**18.1**	**Anwendbarkeit**	
	☐ **0 (nicht anwendbar)** ☐ **1** ☐ **2** ☐ **3** ☐ **4** ☐ **5 (uneingeschränkt anwendbar)**	
**18.2**	**Limitierender Faktor der Anwendbarkeit**	
	☐ **Sprache** ☐ **Inhalt** ☐ **Praktikabilität**	

19 Anwendbarkeit des Items

VISUELL-RÄUMLICHE FÄHIGKEITEN

➢ Bitten Sie den Probanden, die folgenden Buchstaben zu identifizieren.	**Visuell-räumlich**<br>[Punkte 0-4] ☐

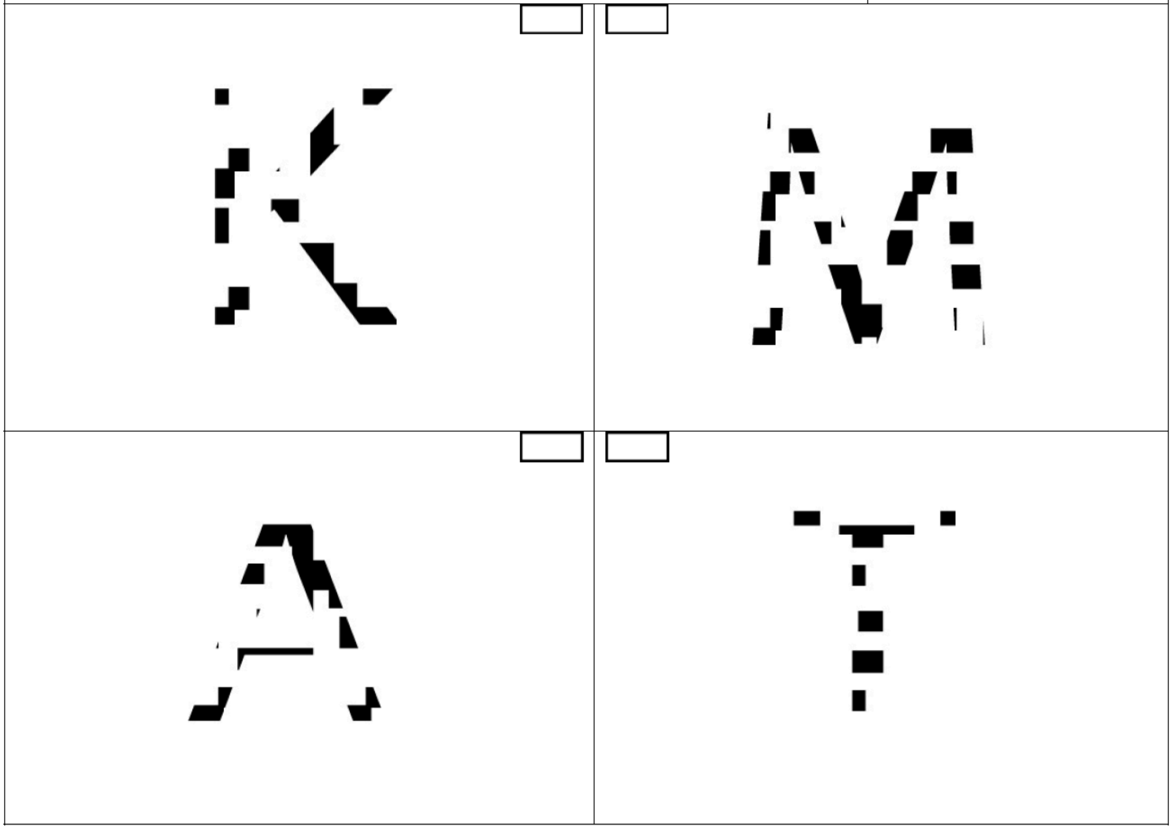

**19.1**	**Anwendbarkeit**
	☐ **0 (nicht anwendbar)** ☐ **1** ☐ **2** ☐ **3** ☐ **4** ☐ **5 (uneingeschränkt anwendbar)**
**19.2**	**Limitierender Faktor der Anwendbarkeit**
	☐ **Sprache** ☐ **Inhalt** ☐ **Praktikabilität**

20 Anwendbarkeit des Items

GEDÄCHTNIS

➢ Fragen Sie: „Können Sie mir den Namen und die Adresse nennen, die wir am Anfang wiederholt haben?"

Peter Müller<br>Dorf Strasse 73<br>Wolfsburg<br>Niedersachsen	………………… ……………………………………<br>…… …………………… ……………………<br>………………………………………………………<br>………………………………………………………	**Gedächtnis**<br>[Punkte 0-7] ☐

**20.1**	**Anwendbarkeit**
	☐ **0 (nicht anwendbar)** ☐ **1** ☐ **2** ☐ **3** ☐ **4** ☐ **5 (uneingeschränkt anwendbar)**
**20.2**	**Limitierender Faktor der Anwendbarkeit**
	☐ **Sprache** ☐ **Inhalt** ☐ **Praktikabilität**

21 Anwendbarkeit des Items

GEDÄCHTNIS

- Der folgende Test sollte nur durchgeführt werden, wenn eines oder mehrere Items der Anschrift nicht korrekt benannt wurden. Wurden alle Items korrekt benannt, überspringen Sie die Aufgabe und vergeben 5 Punkte. Setzen Sie zunächst einen Haken in der schattierten Spalte rechts, bei jedem Item, an das sich der Proband erinnern konnte und vergeben Sie jeweils 1 Punkt.
- Gehen Sie anschließend die nicht erinnerten Items durch und sagen sie dem Probanden „Okay, ich gebe Ihnen eine Hilfestellung: war der Name X, Y oder Z?", usw. Für jedes richtig erkannte Item vergeben Sie ebenfalls 1 Punkt.

Gedächtnis [Punkte 0-5] ☐

Hans Müller		Peter Müller		Peter Schmidt		erinnert	
37		73		76		erinnert	
Dorf Gasse		Land Straße		Dorf Straße		erinnert	
Kassel		Wolfsburg		Braunschweig		erinnert	
Niedersachsen		Sachsen-Anhalt		Baden-Württemberg		erinnert	

**21.1**	**Anwendbarkeit**
	☐ **0 (nicht anwendbar)** ☐ **1** ☐ **2** ☐ **3** ☐ **4** ☐ **5 (uneingeschränkt anwendbar)**
**21.2**	**Limitierender Faktor der Anwendbarkeit**
	☐ **Sprache** ☐ **Inhalt** ☐ **Praktikabilität**

22 Anwendbarkeit der Übersetzung des Addenbrooke's cognitive examination – III Messinstruments

**22.1**	**Anwendbarkeit**
	☐ **0 (nicht anwendbar)** ☐ **1** ☐ **2** ☐ **3** ☐ **4** ☐ **5 (uneingeschränkt anwendbar)**
**22.2**	**Limitierender Faktor der Anwendbarkeit**
	☐ **Sprache** ☐ **Inhalt** ☐ **Praktikabilität**

Viele Dank, dass Sie sich die Zeit genommen haben den Fragebogen auszufüllen!

Ihr ACE-III Studienteam
