# Supplementary material for: Guideline-conform translation and cultural adaptation of the Addenbrooke’s Cognitive Examination III into German
Source: Ger Med Sci. 2020 Apr 6;18:Doc04. doi: 10.3205/000280 (PMC7174851; doi:10.3205/000280)
Supplement: ACE-III Supplement [file GMS-18-04-s-002.pdf]

Supplement Table 1. Cultural adaptations

Category	Page	Translation decisions
Attention	1	Suburb - Stadt: We replaced the item “suburb” with “city”, as this is common practice in Germany
Fluency	1	Letters: We replaced prompting for the letter “C” with the letter “K”, as this letter is more commonly used for language fluency tests in German.
Memory	2	Address: We replaced the name and address from the original publication with a name and address involving common names used in Germany.
Memory	2	Famous people: We replaced premier of NSW with the German “Bundespräsident”, as knowledge of Australian politicians is not usually expected of people residing in Germany.
Language	2/3	Repetition: We replaced the original four words with German words of matching length and complexity. This was not possible via direct translation.
Language	3	Proverbs: The proverbs were replaced by German proverbs matching the length and meaning of the originals.
Language	4	Reading: We chose words matching the length and readability of the original words with a comparable progression in complexity.

List of cultural adaptations that were made during the translation and cultural adaptation process.
