# Supplementary material for: Guideline-conform translation and cultural adaptation of the Addenbrooke’s Cognitive Examination III into German
Source: Ger Med Sci. 2020 Apr 6;18:Doc04. doi: 10.3205/000280 (PMC7174851; doi:10.3205/000280)
Supplement: ACE-III B (German) [file GMS-18-04-s-004.pdf]

ADDENBROOKE'S COGNITIVE EXAMINATION – ACE-III

Deutsche Version B (2012*)

Name:<br>Geburtsdatum:<br>Prob.-Nr. oder Adresse:	Erhebungsdatum: ____/___/___<br>Name des Untersuchenden:_______________________<br>Höchster erreichter Bildungsgrad:__________________<br>Beruf: ____<br>Händigkeit (links/rechts):__________________________

AUFMERKSAMKEIT

➢ Fragen Sie: „Welche/n/s ... haben wir heute?"	Wochentag<br>_________	Datum<br>_________	Monat<br>__________	Jahr<br>__________	Jahreszeit<br>__________	**Aufmerksamkeit** [Punkte 0-5] ☐
➢ Fragen Sie: „In welcher/m ... befinden wir uns?"	Zimmer/ Etage<br>_________	Straße/ Krankenhaus<br>_________	Stadt<br>__________	Bundesland<br>__________	Land<br>__________	**Aufmerksamkeit** [Punkte 0-5] ☐

AUFMERKSAMKEIT

➢ Sagen Sie: „Ich werde Ihnen drei Wörter sagen und bitte Sie, diese danach zu wiederholen: „Apfel, Münze, Stuhl". Nach erfolgter Wiederholung sagen Sie: „Versuchen Sie, sich diese Wörter zu merken, denn ich werde Sie später noch einmal danach fragen."<br>➢ Nur der erste Versuch wird bewertet.<br>➢ Wiederholen Sie bis zu drei Mal, wenn notwendig und notieren Sie die Anzahl der Versuche: _____	**Aufmerksamkeit** [Punkte 0-3] ☐

AUFMERKSAMKEIT

➢ Bitten Sie: „Ziehen Sie von der Zahl 100 jeweils 7 ab, bis ich Sie bitte aufzuhören."<br>➢ Unterbrechen Sie den Probanden bei einem Fehler nicht. Lassen Sie ihn fortfahren und überprüfen die folgenden Antworten (z.B. 93, 84, 77, 70, 63 – 4 Punkte).<br>➢ Stoppen Sie nach 5 Subtraktionen (93, 86, 79, 72, 65): ____ ____ ____ ____ ____	**Aufmerksamkeit** [Punkte 0-5] ☐

GEDÄCHTNIS

➢ Fragen Sie: „Welche drei Wörter hatte ich Sie gebeten zu wiederholen und sich dann zu merken?"<br>______ _______ _______	**Gedächtnis** [Punkte 0-3] ☐

WORTFLÜSSIGKEIT

➢ **Buchstaben**
Sagen Sie: „Ich nenne Ihnen einen Buchstaben und möchte, dass Sie mir so viele Wörter wie möglich sagen, die mit diesem Buchstaben beginnen. Es dürfen aber weder Personen-, noch Ortsnamen sein. Nenne ich Ihnen beispielsweise den Buchstaben „K", wären „Katze, kaufen, kurz" , usw. mögliche Antworten, nicht jedoch „Katharina" oder „Kiel". Haben Sie das verstanden? Sind Sie bereit? Sie haben eine Minute Zeit. Bitte verwenden Sie den Buchstaben „P"."

Wortflüssigkeit [Punkte 0 – 7] ☐

≥ 18	7
14-17	6
11-13	5
8-10	4
6-7	3
4-5	2
2-3	1
0-1	0
Gesamt	korrekt

➢ **Tiere**
Sagen Sie: „Jetzt nennen Sie bitte so viele Tiere wie möglich. Alle Anfangsbuchstaben sind gestattet."

Wortflüssigkeit [Punkte 0 – 7] ☐

≥ 22	7
17-21	6
14-16	5
11-13	4
9-10	3
7-8	2
5-6	1
<5	0
Gesamt	Korrekt

* Deutsche Übersetzung nach modifiziertem FACIT-Protokoll (Eremenco SL et al. Eval Health Prof 2005; 28:212). V 1.0, 12/2016

GEDÄCHTNIS				
➢ Sagen Sie: „Ich werde Ihnen einen Namen und eine Adresse sagen und möchte, dass Sie den Namen und die Adresse wiederholen. Damit Sie sich den Namen und die Adresse besser merken können, wiederholen wir sie drei Mal. Ich frage später noch einmal nach dem Namen und der Adresse."<br><br>➢ Nur der dritte Versuch wird gewertet.				**Gedächtnis**<br>[Punkte 0 – 7]<br>☐
	Erster Versuch	***Zweiter Versuch***	*Dritter Versuch*	
Karin Schmidt<br>Lindenallee 59<br>Freiburg<br>Baden-Württemberg	________ ________<br>____ ______ ______<br>______________<br>______________	________ ________<br>____ ______ ______<br>______________<br>______________	________ ________<br>____ _____ ______<br>______________<br>______________	

GEDÄCHTNIS	
➢ Name des/der amtierenden Bundeskanzlers/in..<br>➢ Name des/der amtierenden Bundespräsidenten/in……………………………………………………………<br>➢ Name des/der amtierenden Präsidenten/in der USA..<br>➢ Name des US-amerikanischen Präsidenten, der in den 1960ern ermordet wurde...................................	**Gedächtnis**<br>[Punkte 0 – 4]<br>☐

SPRACHE	
➢ Legen Sie einen Stift und ein Blatt Papier vor den Probanden. Zur Probe, bitten Sie ihn: „Heben Sie den Stift auf und dann das Blatt Papier." Falls dies nicht gelingt, geben Sie 0 Punkte und beenden Sie diesen Abschnitt.<br>➢ Wurde der Probeversuch korrekt durchgeführt, machen Sie mit den folgenden drei Aufgaben weiter:<br>o "Legen Sie das Blatt Papier auf den Stift"<br>o "Heben Sie den Stift auf, aber nicht das Blatt Papier"<br>o "Reichen Sie mir den Stift, nachdem Sie das Blatt Papier berührt haben"<br>➢ Wichtig: Legen Sie Stift und Papier vor jeder Aufgabe erneut vor den Probanden.	**Sprache**<br>[Punkte 0-3]<br>☐

SPRACHE	
➢ Sagen Sie: „Bitte schreiben Sie zwei Sätze. Der Inhalt der Sätze ist Ihnen überlassen. Ich möchte Sie allerdings bitten, in ganzen Sätzen zu schreiben und Abkürzungen zu vermeiden." Wenn dem Probanden kein Thema einfällt, können Sie Themen vorschlagen: „Sie könnten beispielsweise über Ihren letzten Urlaub, Ihre Hobbies, Ihre Familie oder Kindheit schreiben." Sollte der Proband nur einen Satz aufschreiben, bitten Sie um einen zweiten Satz.<br>➢ Jeder Satz muss sowohl Subjekt als auch Verb enthalten. Grammatik und Rechtschreibung werden bewertet. Die Sätze müssen nicht vom gleichen Thema handeln. Siehe Bewertungsanleitung für weitere Informationen.	**Sprache**<br>[Punkte 0-2]<br>☐

SPRACHE	
➢ Bitten Sie den Probanden folgende Wörter zu wiederholen: „Butterblume", „Ekzentriker", „unentzifferbar", „Statistiker"<br>➢ Vergeben Sie 2 Punkte wenn alle Wörter korrekt wiederholt wurden; 1 Punkt, wenn 3 der Wörter korrekt wiederholt wurden; 0 Punkte bei 2 oder weniger korrekten Wiederholungen.	**Sprache**<br>[Punkte 0-2]<br>☐

SPRACHE

➢ Bitten Sie den Probanden folgendes zu wiederholen: **„Es ist nicht alles Gold, was glänzt.”**	**Sprache** [Punkte 0-1] ☐
➢ Bitten Sie den Probanden folgendes zu wiederholen: **„Der frühe Vogel fängt den Wurm.”**	**Sprache** [Punkte 0-1] ☐

SPRACHE

Sprache [Punkte 0-12] ☐

➢ Bitten Sie den Probanden, folgende Bilder zu benennen:

______ ☐ ______ ☐ ______ ☐

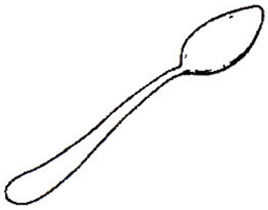
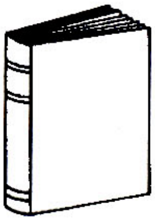
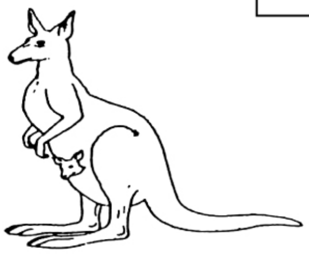

______ ☐ ______ ☐ ______ ☐

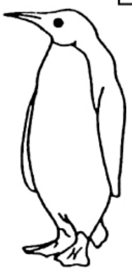
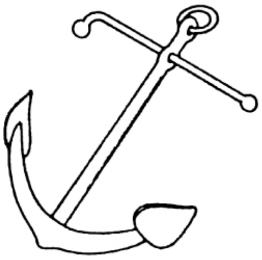
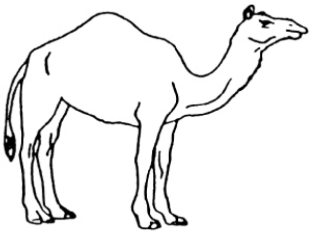

______ ☐ ______ ☐ ______ ☐

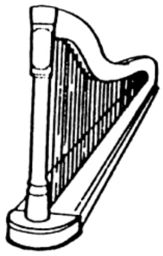
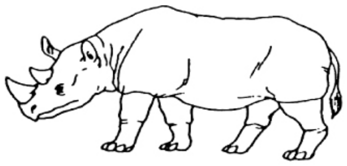
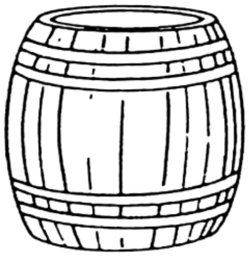

______ ☐ ______ ☐ ______ ☐

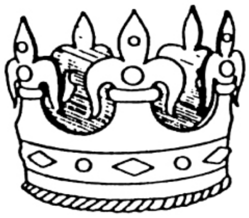
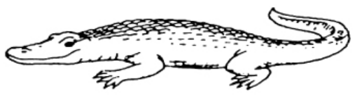
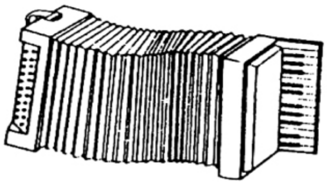

SPRACHE

Sprache [Punkte 0-4] ☐

➢ Mit Hilfe der oben gezeigten Bilder, bitten Sie den Probanden:

- Auf das Bild zu zeigen, das mit Monarchie in Verbindung gebracht werden kann ……………………………………………
- Auf das Bild zu zeigen, das ein Beuteltier darstellt ……………………………………………
- Auf das Bild zu zeigen, welches in der Antarktis gefunden werden kann ……………………………………………
- Auf das Bild zu zeigen, das einen Bezug zur Seefahrt hat ……………………………………………

SPRACHE	
➢ Bitten Sie den Probanden die folgenden Wörter zu lesen: (1 Punkt gibt es sofern alle Wörter korrekt sind)<br><br>**Uhr**<br>**Maß**<br>**fort**<br>**platt**<br>**Schrank**	**Sprache**<br>[Punkte 0-1]<br>☐

VISUELL-RÄUMLICHE FÄHIGKEITEN	
➢ Unendlichkeits-Symbol: Bitten Sie den Probanden, folgendes Symbol nachzuzeichnen.	**Visuell-räumlich**<br>[Punkte 0-1]<br>☐

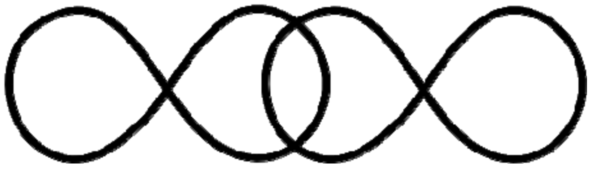

➢ Strichwürfel: Bitten Sie den Probanden, folgende Darstellung nachzuzeichnen (für die Punktevergabe, siehe Bewertungsanleitung).	**Visuell-räumlich**<br>[Punkte 0-2]<br>☐

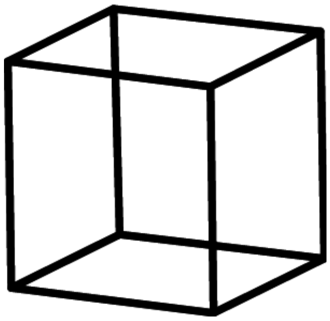

➢ Uhr: Bitten Sie den Probanden, das Ziffernblatt einer Uhr zu zeichnen, deren Zeiger auf Zehn nach Fünf stehen (für Punktevergabe, siehe Nutzerhandbuch: Kreis = 1, Nummern = 2, Zeiger = 2, sofern sie alle korrekt sind).	**Visuell-räumlich**<br>[Punkte 0-5]<br>☐

VISUELL-RÄUMLICHE FÄHIGKEITEN

➢ Bitten Sie den Probanden, die Punkte zu zählen, ohne auf sie zu zeigen.	**Visuell-räumlich**<br>[Punkte 0-4]<br>☐

☐

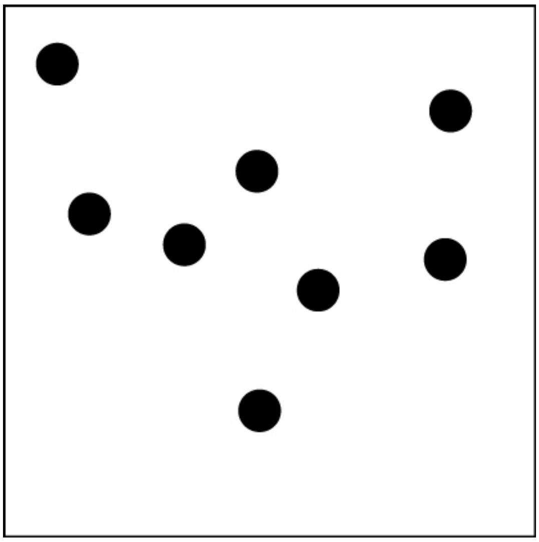

☐

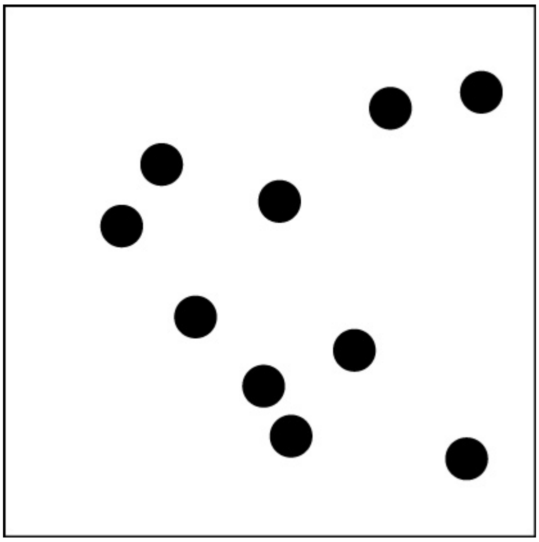

☐

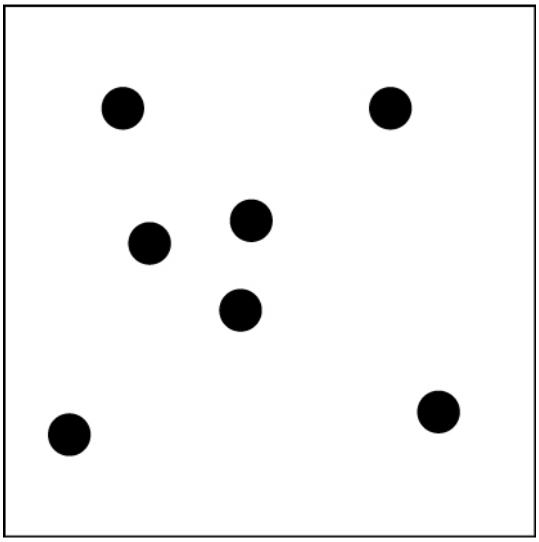

☐

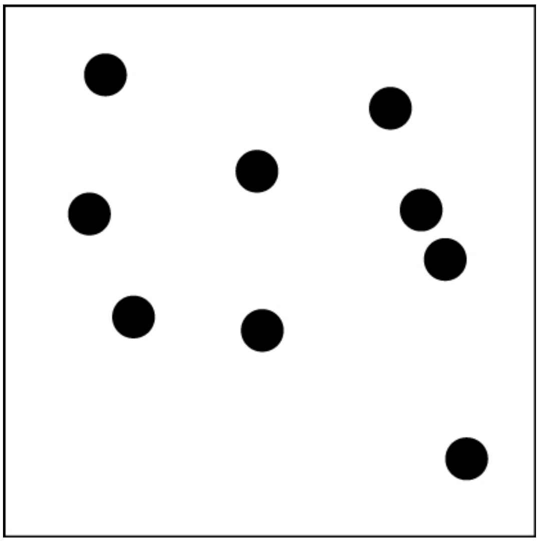

VISUELL-RÄUMLICHE FÄHIGKEITEN

➢ Bitten Sie den Probanden, die folgenden Buchstaben zu identifizieren.	**Visuell-räumlich** [Punkte 0-4] ☐

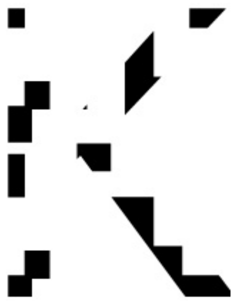

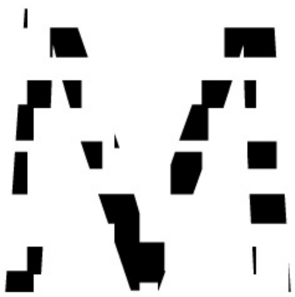

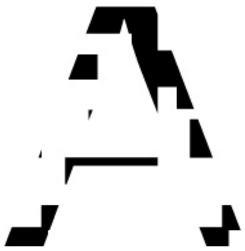

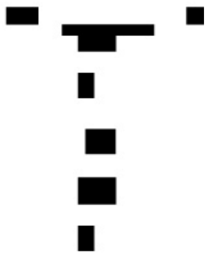

GEDÄCHTNIS

➢ Fragen Sie: „Können Sie mir den Namen und die Adresse nennen, die wir am Anfang wiederholt haben?"

Karin Schmidt	………………… ……………………………………	**Gedächtnis** [Punkte 0-7] ☐
Lindenallee 59	…… ……………………… ………………………	
Freiburg	………………………………………………………	
Baden-Württemberg	…………………………………………………......	

GEDÄCHTNIS

➢ Der folgende Test sollte nur durchgeführt werden, wenn eines oder mehrere Items der Anschrift nicht korrekt benannt wurden. Wurden alle Items korrekt benannt, überspringen Sie die Aufgabe und vergeben 5 Punkte. Setzen Sie zunächst einen Haken in der schattierten Spalte rechts, bei jedem Item, an das sich der Proband erinnern konnte und vergeben Sie jeweils 1 Punkt.<br>➢ Gehen Sie anschließend die nicht erinnerten Items durch und sagen sie dem Probanden „Okay, ich gebe Ihnen eine Hilfestellung: war der Name X, Y oder Z?", usw. Für jedes richtig erkannte Item vergeben Sie ebenfalls 1 Punkt.	**Gedächtnis** [Punkte 0-5] ☐

Karin Schmidt		Sarah Müller		Karin Schuster		erinnert	
39		52		59		erinnert	
Lindenallee		Gartenallee		Lindenstraße		erinnert	
Freiburg		Stuttgart		Frankfurt		erinnert	
Niedersachsen		Sachsen-Anhalt		Baden-Württemberg		erinnert	

GESAMTPUNKTZAHL

**ACE-III Gesamtpunktzahl**	/100
**Aufmerksamkeit**	/18
**Gedächtnis**	/26
**Wortflüssigkeit**	/14
**Sprache**	/26
**Visuell-räumlich**	/16
