# Supplementary material for: Guideline-conform translation and cultural adaptation of the Addenbrooke’s Cognitive Examination III into German
Source: Ger Med Sci. 2020 Apr 6;18:Doc04. doi: 10.3205/000280 (PMC7174851; doi:10.3205/000280)
Supplement: M-ACE C (German) [file GMS-18-04-s-008.pdf]

MINI – ADDENBROOKE'S COGNITIVE EXAMINATION
Deutsche Version C (2014*)

Name:<br>Geburtsdatum:<br>Prob.-Nr. oder Adresse:	Erhebungsdatum: ____/___/___<br>Name des Untersuchenden:________________________<br>Höchster erreichter Bildungsgrad:___________________<br>Beruf: ____<br>Händigkeit (rechts/links): __________________________

AUFMERKSAMKEIT

➢ Fragen Sie: „Welchen/s …haben wir heute?"	Wochentag<br>________	Datum<br>_________	Monat<br>_________	Jahr<br>_________	**Aufmerksamkeit** [Punkte 0-4]

GEDÄCHTNIS

Gedächtnis [Punkte 0 – 7]

- ➢ Sagen Sie: „Ich werde Ihnen einen Namen und eine Adresse sagen und möchte, dass Sie den Namen und die Adresse wiederholen. Damit Sie sich den Namen und die Adresse besser merken können, wiederholen wir sie drei Mal. Ich frage später noch einmal nach dem Namen und der Adresse."
- ➢ Nur der dritte Versuch wird gewertet.

	Erster Versuch	***Zweiter Versuch***	*Dritter Versuch*
Robert Meier	________ _______	________ _______	________ _______
Marktstraße 24	____ ______ ______	____ ______ ______	____ _____ ______
München	______________	______________	______________
Bayern	______________	______________	______________

WORTFLÜSSIGKEIT - TIERE

- ➢ **Tiere**

Sagen Sie: „Nennen Sie mir bitte so viele Tiere wie möglich. Alle Anfangsbuchstaben sind gestattet. Sie haben eine Minute Zeit. Ab jetzt."

Wortflüssigkeit [Punkte 0 – 7]

≥ 22	7
17-21	6
14-16	5
11-13	4
9-10	3
7-8	2
5-6	1
<5	0
Gesamt	Korrekt

* Deutsche Übersetzung nach modifiziertem FACIT-Protokoll (Eremenco SL et al. Eval Health Prof 2005; 28:212). V 1.0, 12/2016

UHR ZEICHNEN

➢ Uhr: Bitten Sie den Probanden, das Ziffernblatt einer Uhr zu zeichnen, deren Zeiger auf Zehn nach Fünf stehen (für Punktevergabe, siehe Nutzerhandbuch : Kreis = 1, Nummern = 2, Zeiger = 2, sofern Sie alle korrekt sind).	**Visuell-räumlich** [Punkte 0-5] ☐

GEDÄCHTNIS - ERINNERN

➢ Fragen Sie: „Können Sie mir den Namen und die Adresse nennen, die wir am Anfang wiederholt haben?”

Robert Meier	....	**Gedächtnis** [Punkte 0-7] ☐
Marktstraße	....	
24 München	....	
Bayern	....	

**Gesamtpunktzahl**	**/ 30**
