# Supplementary material for: Guideline-conform translation and cultural adaptation of the Addenbrooke’s Cognitive Examination III into German
Source: Ger Med Sci. 2020 Apr 6;18:Doc04. doi: 10.3205/000280 (PMC7174851; doi:10.3205/000280)
Supplement: ACE-III Scoring (German) [file GMS-18-04-s-009.pdf]

Anleitung zur Durchführung und Bewertung des ACE-III und M-ACE Deutsche Version – (2014*)

Der Addenbrooke's Cognitive Examination-III (ACE-III) ist ein kurzer Test zur Erfassung von fünf kognitiven Domänen: Aufmerksamkeit, Gedächtnis, Wortflüssigkeit, Sprache und visuell-räumliche Fähigkeiten.

Der ACE-III wurde von Neuroscience Research Australia entwickelt (NeuRA; www.neura.edu.au) und ersetzt seinen Vorgänger, Addenbrooke's Cognitive Examination-Revised.

Die erreichbare Höchstpunktzahl beträgt 100 Punkte, wobei gilt: je höher das Ergebnis desto besser die kognitive Funktion. Die Durchführung des ACE-III nimmt etwa 15 Minuten in Anspruch, seine Auswertung weitere 5.

Der Mini-ACE (M-ACE) - eine Kurzfassung des ACE-III – wurde für Situationen entwickelt, in denen die Durchführung des kompletten ACE-III unpraktisch ist. Die erreichbare Höchstpunktzahl beträgt hierbei 30 Punkte, wobei auch hier gilt: je höher das Ergebnis desto besser die kognitive Funktion.
Die Durchführung des M-ACE dauert etwa 5 Minuten und die Auswertung sollte nicht länger als 1-2 Minuten dauern.

Die vorliegenden Durchführungshinweise dienen der Klärung von Verständnisproblemen in Bezug auf die Fragen und die Auswertung seitens des Erhebenden. Bitte lesen Sie diese aufmerksam durch, bevor Sie den Test durchführen. Falls möglich, vermeiden Sie die Bewertung während der Erhebung. Einen aufmerksamen Teilnehmer könnte das Abhaken von korrekten, bzw. das Durchstreichen von falschen Antworten bei der Antwortfindung beeinflussen/ablenken oder/und zu unnötiger Aufregung führen.

Der ACE-III und M-ACE, sowie Publikations-Updates und Übersetzungen der Scores stehen für Sie zum Download bereit unter: http://www.neura.edu.au/frontier/research

AUFMERKSAMKEIT - Orientierung - Punkte 0 bis 10 — M-ACE

Durchführung: Bitten Sie den Probanden Ihnen den heutigen Wochentag, das Datum (Tag, Monat, Jahr) und die Jahreszeit zu nennen, sowie den Namen des Krankenhauses (bzw. Gebäudes oder der Hausnummer), die Etage (bzw. Zimmernummer oder Straßenname), die Stadt, das Bundesland und das Land.
Bewertung: 1 Punkt für jede korrekte Antwort. Eine Fehlerspanne von ± 2 Tagen ist für die Datumsangabe gestattet (z.B. der fünfte des Monats, wenn heute der siebte ist). Sagt der Proband etwas wie „der 23.03.", bitten Sie ihn den Monatsnamen zu nennen. Befindet sich der Proband in seiner Wohnumgebung, bitten Sie ihn diese zu identifizieren bsp. über den Namen der Wohneinrichtung oder, anstatt über die Etage, über die Zimmerbezeichnung (z.B. Küche, Wohnzimmer, etc.). Handelt es sich um eine einstöckige Einrichtung, können andere Besonderheiten der Wohngegend herangezogen werden.
Befinden sich die Jahreszeiten im Wechsel (z.B. Ende Februar), und der Proband antwortet „Winter", fragen Sie „Könnte es auch eine andere Jahreszeit sein?". Antwortet er mit „Frühling", geben Sie einen Punkt. Keinen Punkt gibt es, wenn die Antwort „Sommer" oder „Herbst" lautet.

Kommt der Proband von außerhalb, kann die Bewertung des Items „Stadt" etwas großzügiger erfolgen. Gibt ein Proband beispielsweise Berlin als Stadt an, können auf Nachfrage auch benachbarte Orte als korrekt bewertet werden (z.B. Königs-Wusterhausen oder Potsdam).

Jahreszeiten: Herbst – September, Oktober, November; Winter – Dezember, Januar, Februar; Frühling – März, April, Mai; Sommer – Juni, Juli, August.
Für aphasische Probanden: Ermöglichen Sie Probanden, ihre Antworten schriftlich zu geben, falls Sie nicht in der Lage sein sollten, sich verbal zu äußern.
Wichtig: In die Bewertung des M-ACE fließen nur Wochentag, Tag, Monat und Jahr ein, nicht aber die Jahreszeit. Die erreichbare Punktzahl liegt hier zwischen 0 und 4.

AUFMERKSAMKEIT - Merken von 3 Gegenstände - Punkte 0 bis 3

Durchführung: Bitten Sie den Probanden 3 Wörter zu wiederholen und sich diese zu merken. Sprechen Sie langsam. Wiederholen Sie die Wörter falls nötig, aber höchstens 3 Mal. Weisen Sie den Probanden unbedingt darauf hin, dass Sie die Gegenstände zu einem späteren Zeitpunkt erneut abfragen werden.
Bewertung: Nur der erste Versuch wird bewertet. Notieren Sie jedoch die Anzahl an Versuchen, die der Proband benötigt um sich alle 3 Wörter zu merken.

AUFMERKSAMKEIT - Substraktion in 7er Schritten - Punkte 0 bis 5

Durchführung: Bitten Sie den Probanden von der Zahl 100 die Zahl 7 abzuziehen, notieren Sie die Antwort und bitten ihn wiederum 7 von jedem erreichten Ergebnis abzuziehen, bis Sie ihn dann bitten aufzuhören. Beenden Sie die Rechenaufgabe nach 5 Subtraktionsschritten.
Bewertung: Notieren Sie die Antworten und unterbrechen Sie den Probanden nicht nach einem Fehler. Lassen Sie ihn fortfahren und überprüfen Sie die Folgerechnungen auf ihre Richtigkeit (z.B. 92, **85**, 79, **72**, **65** – Punkte = 3).

* Deutsche Übersetzung nach modifiziertem FACIT-Protokoll (Eremenco SL et al. Eval Health Prof 2005; 28:212). V 1.0, 12/2016

GEDÄCHTNIS - Erinnern von 3 gemerkten Gegenständen - Punkte 0 bis 3

Durchführung: Bitten Sie den Probanden, die anfangs gemerkten Objekte zu nennen.
Bewertung: Notieren Sie die Antworten und vergeben Sie je 1 Punkt für jede korrekte Antwort. Geben Sie dem Probanden keine Hilfestellung.

WORTFLÜSSIGKEIT - Buchstaben - Punkte 0 bis 7

Durchführung: Sagen Sie zum Probanden: „Ich nenne Ihnen einen Buchstaben und möchte, dass Sie mir so viele Wörter wie möglich sagen, die mit diesem Buchstaben beginnen. Es dürfen aber weder Personen-, noch Ortsnamen sein. Nenne ich Ihnen beispielsweise den Buchstaben „K", wären „Katze, kaufen, kurz" , usw. mögliche Antworten, nicht jedoch „Katharina" oder "Kiel". Haben Sie das verstanden? Sind Sie bereit?
Sie haben eine Minute Zeit. Bitte verwenden Sie den Buchstaben „P"."
Bewertung: Notieren Sie zuerst die Gesamtzahl der genannten Wörter und zählen im Anschluss die Gesamtzahl korrekt genannter Wörter. Nicht gezählt werden (1) Wiederholungen, (2) Konjugationen oder Deklinationen (z.B. zahlt, zahlen, zahlte – **Punkte** = 1), (3) Falschnennungen (Wörter, die mit einem anderen Buchstaben beginnen), (4) Eigennamen und (5) Plurale (z.B. Vase, Vasen – gesamt = 2, korrekt = 1). Benutzen Sie die dem ACE-III beigefügte Bewertungstabelle um eine Gesamtwertung zu erhalten.

WORTFLÜSSIGKEIT - TIERE - Punkte 0 bis 7 M-ACE

Durchführung: Sagen Sie: „Nennen Sie mir bitte so viele Tiere wie möglich. Alle Anfangsbuchstaben sind gestattet."
Bewertung: Notieren Sie die Gesamtzahl der genannten Tiere. Anschließend zählen Sie die korrekt genannten Wörter. Dies beinhaltet nicht die Nennung von übergeordneten Kategorien, wenn spezifische Arten genannt wurden (z.B. „Fisch", gefolgt von „Lachs" und „Barsch" – gesamt = 3; korrekt = 2).
Die Nennung von verschiedenen Geschlechtern derselben Tierart wird als 1 Punkt bewertet (z.B. „Reh", gefolgt von „Hirsch" wird mit 1 Punkt bewertet).) Es bestehen keinerlei Einschränkungen in der Art von genannten Tieren. Insekten, auch Menschen, prähistorische und ausgestorbene Tiere, sowie mythische Wesen (z.B. Einhorn) sind zulässig.
Sollte der Proband die Fragestellung missverstanden haben und nur Tiere, deren Name mit dem Buchstaben „P" beginnt (z.B. Panda, Possum) aufzählen, weisen Sie ihn darauf hin, dass Tiere mit sämtlichen Anfangsbuchstaben gestattet sind.

WORTFLÜSSIGKEIT - Anterogrades Gedächtnis - Name und Adresse - Punkte 0 bis 7 Durchführung: M-ACE

Durchführung: Sagen Sie: „Ich werde Ihnen einen Namen und eine Adresse sagen und möchte, dass Sie den Namen und die Adresse wiederholen. Damit Sie sich den Namen und die Adresse besser merken können, wiederholen wir sie drei Mal. Ich frage später noch ein Mal nach dem Namen und der Adresse."
Beginnt der Proband frühzeitig, Ihnen nachzusprechen, bitten Sie ihn zu warten, bis Sie die gesamte Anschrift vorgelesen haben.
Bewertung: Notieren Sie die Antworten aus jedem Versuch, wobei jedoch lediglich der dritte Versuch bewertet wird (0-7 Punkte).

GEDÄCHTNIS – Retrogrades Gedächtnis – Bekannte Persönlichkeiten – Punkte 0 bis 4

Durchführung: Bitten Sie den Probanden, Ihnen folgende Namen zu nennen: Den Namen des amtierenden Bundeskanzlers, des amtierenden Bundespräsidenten, des Präsidenten der Vereinigten Staaten von Amerika sowie des Präsidenten der Vereinigten Staaten von Amerika, der in den 1960ern ermordet wurde.

Bewertung: Jede korrekte Antwort wird mit einem Punkt bewertet. Nachnamen sind erlaubt (z.B. „Merkel"). Erfragen Sie den Nachnamen, sollte nur der Vorname genannt werden (z.B. „Angela"). Ist der vollständig genannte Name falsch (z.B. „Helmut Kohl"), werden 0 Punkte vergeben. Erfolgte kürzlich ein Amtswechsel, erfragen Sie den Namen des ehemaligen Amtsinhabers.

SPRACHE – Verständnis – Punkte 0 bis 3

Durchführung:
Legen Sie einen Stift und ein Blatt Papier vor den Probanden. Zur Probe, bitten Sie ihn: „Heben Sie den Stift auf und dann das Blatt Papier." Falls dies nicht gelingt, geben Sie 0 Punkte und beenden Sie diesen Abschnitt.
Sonst fahren Sie mit den 3 Anweisungen aus dem Fragebogen fort. Stift und Papier werden vor jeder Anweisung erneut vor den Probanden gelegt.

Bewertung: Jede korrekt ausgeführte Anweisung wird mit 1 Punkt bewertet

SPRACHE – Sätze Schreiben – Punkte 0 bis 2

Durchführung: Bitten Sie den Probanden, 2 Sätze aufzuschreiben. Schlagen Sie dabei einige Themen vor (z.B. Urlaubsreisen, Hobbies, die Familie oder die Kindheit), wenn es ihm schwer fallen sollte, der Aufforderung zu folgen. Sollte der Proband nur einen Satz zu Papier bringen, bitten Sie ihn unbedingt, einen zweiten Satz zu schreiben.
Bewertung: Jeder Satz muss sowohl Subjekt als auch Verb enthalten. Grammatik und Rechtschreibung werden bewertet. Die Sätze müssen sich inhaltlich nicht auf das gleiche Thema beziehen. Sollte der Proband auch nach Aufforderung nicht in der Lage sein, einen zweiten Satz zu notieren, gibt es hierfür Punktabzug.

Punkte-verteilung	Satzbeschreibung
**2**	Zwei grammatikalisch korrekte Sätze ohne Rechtschreibfehler. Die Sätze müssen nicht zwingend das gleiche Thema haben.<br><br>z.B. „Ich besuche gerne den Strand. Ich habe drei Enkelkinder.”
**1**	Zwei vollständige Sätze, jedoch mit Fehlern in der Grammatik und/oder Rechtschreibung.<br>Ein einzelner grammatikalisch korrekter Satz ohne Rechtschreibfehler.<br><br>z.B. „Ich besuche gerne den Strand. Ich tanze auch gerne.”<br>„Ich gerne Strand. Ich auch tanzen.”<br>„Ich schwimme gerne.”
0	Ein einzelner Satz mit Fehlern in der Grammatik und/oder Rechtschreibung.<br>Einige Wörter mit losem Zusammenhang (z.B. „mag tanzen”), ein Ort (z.B. „Charité Krankenhaus”) oder der Name einer Person.<br>Kein Satz konnte zu Papier gebracht werden.

SPRACHE – Wiederholung einzelner Wörter – Punkte 0 bis 2

Durchführung: Bitten Sie den Probanden, Ihnen jedes Wort nachzusprechen, wobei Sie jeweils nur ein Wort nennen.
Bewertung: Klingt die Aussprache nicht normal (z.B. stockend, forciert, undeutlich) wird das Wort als falsch bewertet. Nur der jeweils erste Versuch wird bewertet.
Vergeben Sie 2 Punkte, wenn alle Wörter korrekt wiederholt wurden; 1 Punkt, wenn 3 der Wörte korrekt wiederholt wurden; 0 Punkte bei 2 oder weniger korrekten Wiederholungen.

SPRACHE – Sprichwörter – Punkte 0 bis 2

Durchführung: Bitten Sie den Probanden, jedes Sprichwort nachzusprechen.
Bewertung: Nur teilweise korrekte Wiederholungen werden nicht akzeptiert (z.B. „Es ist nicht alles Gold was glitzert”). Jedes Sprichwort zählt 1 Punkt.

Wichtig: Hat der Proband ein Sprichwort wiederholt, können Sie ihn fragen: „Was bedeutet dieses Sprichwort?” oder „Wie würden Sie dieses Sprichwort jemandem erklären, der es noch nie zuvor gehört hat?” Diese Ergänzung ermöglicht dem Kliniker eine kurze Beurteilung des Abstraktionsvermögens des Probanden.

SPRACHE – Objektbenennung – Punkte 0 bis 12

Durchführung: Bitten Sie den Probanden, jedes der ihm gezeigten Bilder zu benennen.
Bewertung: Richtige Antworten sind: Löffel, Buch, Känguru, Pinguin, Anker, Kamel / Dromedar, Harfe, Nashorn, Fass / Tonne, Krone, Krokodil / Alligator, Akkordeon / Handharmonika / Quetschkommode.
Pro Bild wird ein Punkt vergeben.

SPRACHE – Verständnis – Punkte 0 bis 4

Durchführung: Bitten Sie den Probanden auf die Bilder zu zeigen, die der vorgelesenen Aussage entsprechen.
Geben Sie keinerlei Rückmeldung bezüglich der Wortbedeutung.
Bewertung: 1 Punkt für jedes Bild. Selbstverbesserungen sind erlaubt.

SPRACHE – Lesen – Punkte 0 oder 1

Durchführung: Bitten Sie den Probanden, die Wörter laut vorzulesen.
Bewertung: 1 Punkt wird bewertet, wenn alle fünf Wörter korrekt vorgelesen wurden. Notieren Sie Fehler mit Hilfe des phonetischen Alphabets, wenn möglich.

VISUELL-RÄUMLICHE FÄHIGKEITEN – Überschneidende Unendlichkeitssymbole – Punkte 0 oder 1

Durchführung: Bitten Sie den Probanden, die sich überschneidenden Unendlichkeitssymbole nachzuzeichnen.
Bewertung: Es wird ein Punkt gegeben, wenn zwei Unendlichkeitssymbole gezeichnet werden, die sich außerdem überschneiden. Beide Symbole müssen sich überkreuzen und dürfen nicht wie einfache Kreise aussehen.

Punkte = 0

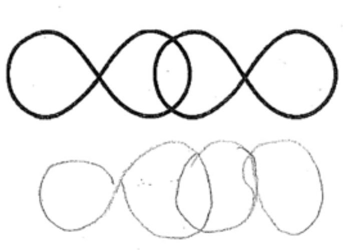
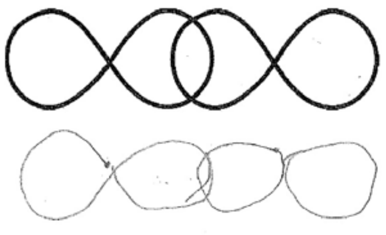
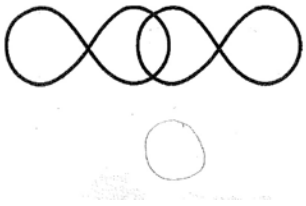

Punkte = 1

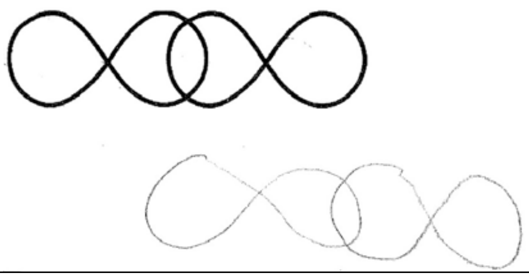
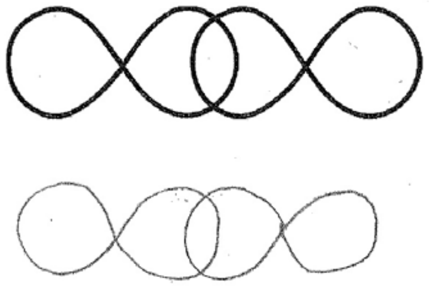

VISUELL-RÄUMLICHE FÄHIGKEITEN - 3D Strichwürfel - Punkte 0 bis 2

Durchführung: Bitten Sie den Probanden den 3D Strichwürfel nachzuzeichnen.
Bewertung: Damit 2 Punkte gegeben werden können, sollte der Würfel aus 12 Linien bestehen, auch bei abweichenden Proportionen. 1 Punkt wird gegeben, wenn der Würfel aus weniger als 12 Linien besteht aber die allgemeine Würfelform gewahrt ist.

Punkte = 1

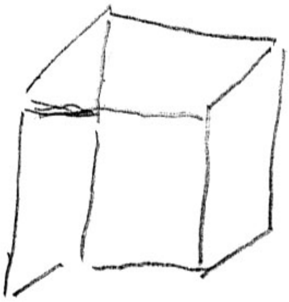
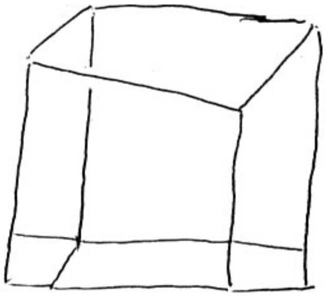
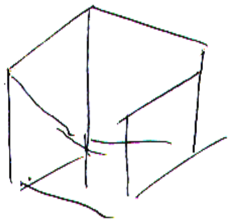

Punkte = 2

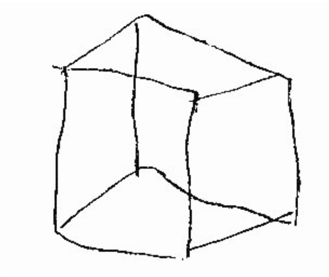
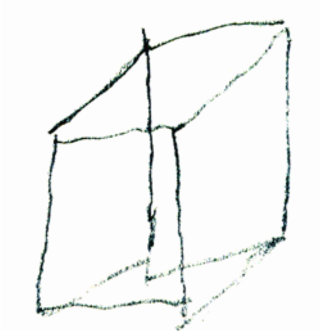
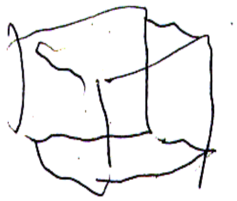
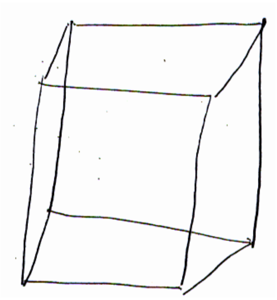

VISUELL-RÄUMLICHE FÄHIGKEITEN – Uhr – Punkte 0 bis 5 M-ACE

Durchführung: Bitten Sie den Probanden, das Ziffernblatt einer Uhr zu zeichnen, deren Zeiger auf Zehn nach Fünf stehen. Ist der Proband mit seiner ersten Zeichnung unzufrieden, kann ihm ein zweiter Versuch gewährt werden, wobei dann nur der zweite Versuch bewertet wird. Fallen dem Probanden während des Zeichnens Fehler auf, kann er diese korrigieren.
Bewertung: Im Folgenden werden die Bewertungskriterien für die 5 möglichen Punkte gezeigt.

**Kreis**	Maximal 1 Punkt für einen akzeptablen Kreis
**Zahlen**	2 Punkte, wenn alle Zahlen gleichmäßig im Kreis verteilt wurden. Eine leichte Drehung des gesamten Ziffernblattes ist gestattet.<br>1 Punkt, wenn zwar alle Zahlen eingezeichnet wurden, diese sich aber entweder außerhalb des Kreises befinden, oder innerhalb des Kreises unregelmäßig verteilt sind.<br>0 Punkte, wenn nicht alle Zahlen eingezeichnet wurden.
**Zeiger**	2 Punkte wenn beide Zeiger mit korrekten Längenverhältnissen eingezeichnet sind und die richtige Uhrzeit anzeigen (Sie können den Probanden fragen, welcher Zeiger der große und welcher der kleine sein soll).<br>1 Punkt, wenn die Zeiger die Richtige Uhrzeit anzeigen, die Längenverhältnisse der Zeiger aber nicht stimmen.

1 Punkt, wenn zwar beide Zeiger eingezeichnet wurden, aber nur ein Zeiger mit richtiger Länge die richtige Zeit anzeigt.
0 Punkte, wenn beide Zeiger eingezeichnet wurden, aber mit falschen Längenverhältnissen und nur einer davon die richtige Zeit anzeigt.
0 Punkte, wenn zwar beide Zeiger eingezeichnet wurden, aber mit falschen Längenverhältnissen und die falsche Uhrzeit anzeigen.
0 Punkte, wenn nur ein Zeiger eingezeichnet wurde.

**Punkte 1**	**Punkte 2**	**Punkte 2**
Kreis (1); es ist nicht sicher erkennbar, ob alle Zahlen vorhanden sind (0); unklare Platzierung der Zeiger 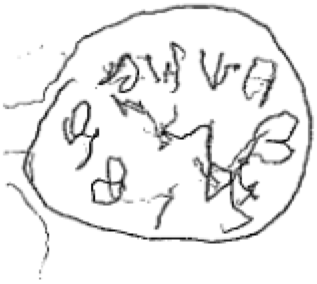	Kreis (1); ein Zeiger mit korrekter Länge zeigt die richtige Zeit an(1) 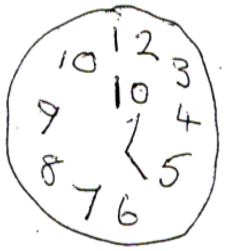	Kreis (1); alle Zahlen sind zwar vorhanden, befinden sich aber nicht alle innerhab des Kreises (1) 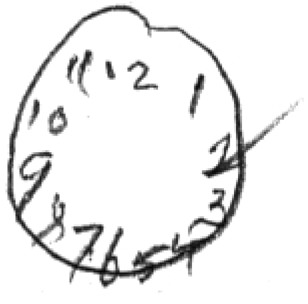
**Punkte 2**	**Punkte 3**	**Punkte 3**
Kreis (1); alle Zahlen, die sich aber nicht alle innerhalb des Kreises befinden (1); zwei Zeiger, von denen zwar einer die richtige Zeit zeigt, aber beide Zeiger gleich lang sind (0) 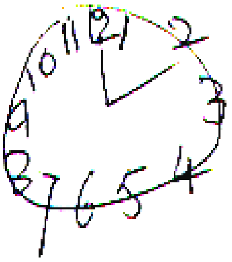	Kreis (1); alle Zahlen sind vorhanden und gleichmäßig verteilt (leichte Drehung des Zifferblattes ist akzeptabel) (2); nur ein Zeiger (0) 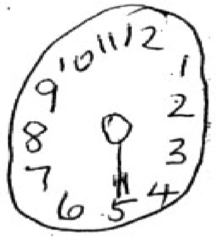	Kreis (1); Zahlen liegen nicht innerhalb des Kreises und die 10 kommt zwei Mal vor (0); Zeiger sind korrekt platziert und haben das korrekte Längenverhältnis (2) 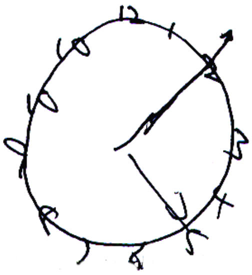
**Punkte 3**	**Punkte 4**	**Punkte 4**
Kreis (1); Zahlen sind ungleichmäßig eingezeichnet (1); ein Zeiger ist korrekt platziert und hat die korrekte Länge (1) 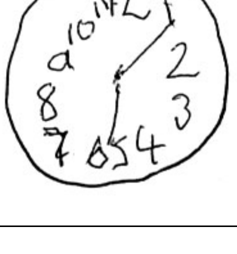	Kreis (1); Zahlen sind vollständig, aber nicht gleichmäßig verteilt (1); beide Zeiger sind korrekt platziert und haben die richtige Länge (2) 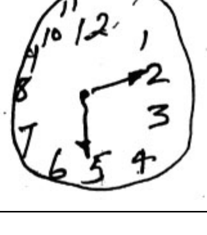	Kreis (1); Alle Zahlen sind gleichmäßig verteilt (2); ein Zeiger ist korrekt platziert und hat die richtige Länge (1) 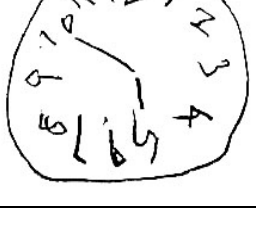

Punkte 5

Kreis (1); Alle Zahlen sind gleichmäßig verteilt (2); beide Zeiger sind korrekt platziert (2)

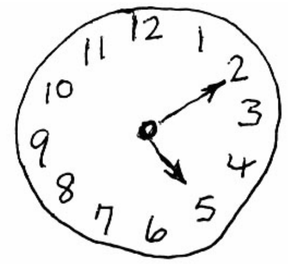

WAHRNEHMUNG – Punkte zählen – Punkte 0 bis 4

Durchführung: Fragen Sie den Probanden, wie viele Punkte sich in jedem Kasten befinden. Er darf dabei nicht mit dem Finger auf die Punkte zeigen.
Bewertung: 1 Punkt für jede richtige Antwort. Richtige Antworten: 8, 10, 7 und 9.

WAHRNEHMUNG – Buchstaben erkennen – Punkte 0 bis 4

Durchführung: Bitten Sie den Probanden, Ihnen die Buchstaben zu nennen, die in dem jeweiligen Kasten zu erkennen sind. Hierbei darf mit dem Finger auf das Blatt gezeigt werden.
Bewertung: 1 Punkt für jede richtige Antwort. Richtige Antworten: K, M, A und T.
Für aphasische Probanden: Ist der Proband nicht im Stande, die Zahl der Punkte oder den Namen des Buchstabens zu nennen, kann er die Antwort auch aufschreiben. Im Falle der Buchstaben ist auch ein dem Buchstaben entsprechender Laut als Antwort zulässig (z.B. „mmm”).

GEDÄCHTNIS – Erinnern von Name und Adresse – Punkte 0 bis 7 M-ACE

Durchführung: Fragen Sie den Probanden: „Können Sie mir den Namen und die Adresse nennen, die wir am Anfang wiederholt haben?”
Bewertung: 1 Punkt für jedes erinnerte Item, entsprechend der Punkteliste im Fragebogen.

Peter Müller
Dorf Strasse 73
Wolfsburg
Niedersachsen

Beispiel: 1a

Peter Meier<br>Dorf Strasse 78<br>Freiburg<br>….	1 + 0<br>0 + 1 + 1<br>0<br>0	**Punkte 3/7**

Beispiel: 2a

Peter Müller<br>Land Straße 73<br>….<br>Niedersachsen	1 + 1<br>1 + 0 + 1<br>0<br>1	**Punkte 5/7**

Beispiel: 3a

Peter Meier<br>Land Weg 33<br>Land Straße<br>Hannover<br>Niedersachsen	1 + 0<br>0 + 0 + 0<br>0 + 0<br>0<br>1	**Punkte 2/7**

GEDÄCHTNIS – Erkennen von Name und Adresse – Punkte 0 bis 5

Durchführung: Diese Aufgabe wird Probanden gegeben, falls sie sich an ein oder mehr Items aus der vorangegangenen Aufgabe nicht erinnern können.
Sie bietet den Probanden die Möglichkeit, die Items zu erkennen, an die er sich nicht erinnern konnte.
Wurden alle Items korrekt benannt, überspringen Sie die Aufgabe und vergeben 5 Punkte.
Viele Probanden erinnern sich jedoch nur an Teile des Namens und der Adresse.
Setzen Sie zunächst einen Haken in der schattierten Spalte rechts, bei jedem Item, an das sich der Proband erinnern konnte und vergeben Sie jeweils 1 Punkt. Gehen Sie anschließend die nicht erinnerten Items durch und sagen sie dem Probanden „*Okay, ich gebe Ihnen eine Hilfestellung: war der Name X, Y oder Z?*", usw.
Bewertung: Für jedes richtig erkannte Item vergeben Sie ebenfalls 1 Punkt. Somit sind in dieser Aufgabe maximal 5 Punkte zu erreichen.

Beispiel 1b (basierend auf Beispiel 1a)

Tester hakt „Dorf Strasse" in der schattierten rechten Spalte ab, weil der Proband dieses Item korrekt erinnert hat. Dann sollte der Proband gefragt werden:<br>• Hieß er Hans Müller, Peter Müller oder Peter Schmidt?<br>• War die Hausnummer die 37, 73 oder die 76?<br>• War die Stadt Kassel, Wolfsburg oder Braunschweig?<br>• War das Bundesland Niedersachsen, Sachsen-Anhalt oder Baden-Württemberg?	Die Antwort des Probanden:<br><br>Peter Müller<br>76<br>Wolfsburg<br>Sachsen-Anhalt	1<br>0<br>1<br>0<br>+ 1 (Dorf Strasse)<br>**Punkte 3/5**

Beispiel 2b (basierend auf Beispiel 2a)

Tester hakt „Peter Müller", „73" and „Niedersachsen" in der schattierten rechten Spalte ab, weil der Proband diese Items korrekt erinnert hat. Dann sollte der Proband gefragt werden:<br>• War es die Dorf Gasse, Land Straße oder die Dorf Strasse?<br>• War die Stadt Kassel, Wolfsburg oder Braunschweig?	Die Antwort des Probanden:<br><br>Dorf Strasse<br>Wolfsburg	1<br>1<br>+ 3 (Peter Müller, 73, Niedersachsen)<br>**Score 5/5**

Beispiel 3b (basierend auf Beispiel 3a)

Tester hakt „Niedersachsen" in der schattierten rechten Spalte ab, weil der Proband dieses Item korrekt erinnert hat. Dann sollte der Proband gefragt werden:<br>• Hieß er Hans Müller, Peter Müller oder Peter Schmidt?<br>• War die Hausnummer die 37, 73 oder die 76?<br>• War es die Dorf Gasse, Land Straße oder die Dorf Strasse?<br>• War die Stadt Kassel, Wolfsburg oder Braunschweig?	Die Antwort des Probanden:<br><br>Hans Müller<br>37<br>Landstraße<br>Braunschweig	0<br>0<br>0<br>0<br>+1 (Niedersachsen)<br>**Score 1/5**

PUNKTE – Abschnitt- und Gesamtscores für den ACE-III und M-ACE

Bewertung: Addieren Sie nun alle Punkte für die fünf Abschnitte (Aufmerksamkeit, Gedächtnis, Wortflüssigkeit, Sprache, visuell-räumlich), um die jeweiligen Abschnitts-Scores für den ACE-III zu erhalten. Der Gesamtscore für den ACE-III (/100) ergibt sich aus der Summe der fünf Abschnitts-Scores.
